# Localization and Characterization of Major Neurogenic Niches in the Brain of the Lesser-Spotted Dogfish *Scyliorhinus canicula*

**DOI:** 10.3390/ijms24043650

**Published:** 2023-02-11

**Authors:** Sara Bagnoli, Elena Chiavacci, Alessandro Cellerino, Eva Terzibasi Tozzini

**Affiliations:** 1 Biology Laboratory (BIO@SNS), Scuola Normale Superiore, 56126 Pisa, Italy; 2 Biology and Evolution of Marine Organisms Department (BEOM), Stazione Zoologica Anton Dohrn, 80121 Napoli, Italy; 3 Fritz Lipmann Institute for Age Research, Leibniz Institute, 07745 Jena, Germany

**Keywords:** adult neurogenesis, dogfish, catshark, sharks, neurogenic niches, radial glia, PCNA, S100β

## Abstract

Adult neurogenesis is defined as the ability of specialized cells in the postnatal brain to produce new functional neurons and to integrate them into the already-established neuronal network. This phenomenon is common in all vertebrates and has been found to be extremely relevant for numerous processes, such as long-term memory, learning, and anxiety responses, and it has been also found to be involved in neurodegenerative and psychiatric disorders. Adult neurogenesis has been studied extensively in many vertebrate models, from fish to human, and observed also in the more basal cartilaginous fish, such as the lesser-spotted dogfish, *Scyliorhinus canicula,* but a detailed description of neurogenic niches in this animal is, to date, limited to the telencephalic areas. With this article, we aim to extend the characterization of the neurogenic niches of *S. canicula* in other main areas of the brain: we analyzed via double immunofluorescence sections of telencephalon, optic tectum, and cerebellum with markers of proliferation (PCNA) and mitosis (pH3) in conjunction with glial cell (S100β) and stem cell (Msi1) markers, to identify the actively proliferating cells inside the neurogenic niches. We also labeled adult postmitotic neurons (NeuN) to exclude double labeling with actively proliferating cells (PCNA). Lastly, we observed the presence of the autofluorescent aging marker, lipofuscin, contained inside lysosomes in neurogenic areas.

## 1. Introduction

Neurogenesis is the process by which the cells of the adult nervous system are generated during development to give rise to neurons that constitute the adult brain. Neurogenesis is strongly reduced after development, but does not cease completely, and studies of the last 40 years have demonstrated that the persistence of embryonic neurogenic niches in at least some circumscribed regions of the adult brain is a basal trait of gnathostomes [1,2,3,4,5], whereas it seems to be absent in cyclostomes [6,7]. A large body of studies investigated the origin, organization, regulation, and function of adult neurogenic niches in the rodent brain. In rodents, neurogenesis persists in two restricted pallial regions: the subventricular zone (SVZ) in the subpallium and the dentate gyrus (DG) of the hippocampus in the pallium. The neurogenic activity of these two regions is sustained by stem cells of glial nature (radial glia, RG), which have embryonic features and embryonic origin, and the molecular signature of the hippocampal neurogenic process in adults is de facto identical to the process that generates hippocampal neurons during the perinatal phase [8,9,10,11]. More recently, adult neurogenesis was also described in the rodent hypothalamus and in the striatum of primates [12,13]. The SVZ is the most active zone, and it produces dopaminergic, GABAergic, and some glutamatergic neurons of the olfactory bulb. Its activity is necessary for the homeostasis of the olfactory bulb: deletion of adult progenitors leads to its degeneration, and enhancement of adult neurogenesis improves odor discrimination [14,15]. The subventricular zone is structured in a medial, a lateral, and a dorsal part. Stem cells of the three subdivisions of the SVZ are originated during embryonic life and are in clonal relationships with cortical neurons (dorsal division), septal neurons (medial division), and striatal neurons (lateral division), even though all three divisions produce neurons of the olfactory bulb in adulthood [16]. The RG of the rodent dentate gyrus is located in the subgranular zone (SGZ). The SGZ produces neurons that remain in the dentate gyrus; the neurogenic output of the SGZ is much smaller than that of the SVZ [17] and decays exponentially with age [18]. In rodents, hippocampal adult neurogenesis is important for some specific cognitive functions, such as pattern discrimination [19]. In humans, it is still a matter of debate whether adult hippocampal neurogenesis persists after adolescence [20,21,22,23]. Loss of adult neurogenesis was also reported in cetaceans [24]. Adult neurogenesis in the hippocampus appears as a basal trait of mammals, but its age-dependent decline does not scale with lifespan, and, therefore, it is strongly reduced in adults of long-living species [25].

RG cells may either divide symmetrically (self-renewal mode) or asymmetrically, giving rise to a transient amplifying progenitor (neurogenic mode). The control between these two modes is finely tuned by electrical activity [26,27].

In teleost fish, as opposed to mammals, adult neurogenesis is widespread, and more than ten neurogenic niches were described along the rostro-caudal axis of the cerebral ventricle, whereas some regions, such as the subpallium, optic tectum, and cerebellum, are notable for a particularly high mitotic activity [28]. Some of these neurogenic niches contain cells that are positive for the expression of glial markers and with clear RG morphology. This is well-described in the pallium of zebrafish, where RG sustains neurogenesis via the production of transient progenitors, as in mammals [29]. The subpallial niche of teleost fishes, on the other hand, contains stem cells devoid of glial markers that show high-mitotic activity. In teleost fishes, the lateral part of the pallium, the marginal zone of the optic tectum, and the cerebellum contain highly active neuroepithelial cells, whereas the mitotic activity of the radial glia in these structures is low (optic tectum) or negligible (cerebellum) [30,31,32]. Nonglial, possibly neuroepithelial cells, were also described in the pallium of the adult turquoise killifish as a possible adaptation to sustain the explosive post-hatch growth of this annual fish [33]. Is widespread adult neurogenesis a basal vertebrate trait, or a derived trait of teleost fishes? This question is further complicated by the derived morphogenesis pattern of the teleost telencephalon with an eversion of the telencephalic vesicles that lead to the dorsal displacement of the ventricle itself, with dorsal displacement of the pallial RGs [34]. To answer this question, a systematic analysis of adult neurogenesis in a basal vertebrate clade is mandatory.

Chondrichthyans represent the basal clade of gnathostomes. The morphogenesis of the chondrichthyan brain is characterized by the same evagination of telencephalic vesicles that characterizes the brain morphogenesis in tetrapods; therefore, the chondrichthyan brain possesses two lateral ventricles, in contrast to the peculiar eversion process that characterizes the embryonic development of the ray-finned fish telencephalon [35]. Interestingly, the cephalic index of chondrichthyans is larger than in teleosts and more similar to that of birds and mammals, and the allometric scaling between the different brain divisions is identical to primates, making neurobiological investigations in this class particularly interesting [36,37]. The spotted catshark, *Scyliorhinus canicula*, is a small (<1 m) galeomorph shark that is common in the European North Atlantic and the Mediterranean Sea. Due to ease of collection and possibility of captive breeding, this species has become a model chondrichthyan. The patterns of embryonic neurogenesis in the spotted catshark *Scyliorhinus canicula* were described in a series of papers that analyzed embryonic neurogenesis in the telencephalon and the cerebellum, and adult neurogenesis was demonstrated in the adult telencephalon and retina with a clear, age-dependent decline, at least in the retina [38,39,40,41,42,43,44,45]. The analysis of the adult telencephalon, in particular, demonstrated the existence of a neurogenic niche lining the entire third ventricle and confirmed the presence of mitotically active RG cells, indicating that the organization of the telencephalic niche is similar to the SVZ of tetrapods. Contrary to teleosts, a subpallial niche containing nonglial progenitors could not be identified. Yet, a systematic analysis of adult neurogenesis in this model is still missing. A particular focus of interest is the cerebellum, as chondrichthyans evolved a particularly complex cerebellar structure, with foliation and expansion of the region receiving inputs from the VIII cranial nerve [46].

In the present study, we used immunohistochemical and in situ hybridization techniques to characterize the putative neurogenic niches along the entire rostro-caudal axis of *S. canicula*. We used immunoreactivity for S100β as a prototypical glial marker, the RNA-binding protein Musashi-1 as a general marker of neuronal progenitors, β-III Tubulin as a marker of newly differentiated neuronsand PCNA and phospho-H3 as mitotic markers, in order to differentiate regions containing glial and nonglial progenitors.

Moreover, we analyzed a specific aspect of adult neurogenesis: the presence of protein aggregates in the lysosome. A recent study in mice demonstrated that quiescent adult neural stem cells contain enlarged lysosomes filled with protein aggregates [47]. This process is reversible, and clearance of aggregates activates the stem cells. The presence of such aggregates in other species has not been investigated so far; therefore, we decided to study this phenomenon in the adult stem cells of the catshark.

## 2. Results

In the present work, we analyzed the neurogenic niches present in different areas of the brain of the lesser-spotted dogfish, *Scyliorhinus canicula.*

During development, the brain of *S. canicula* follows an evaginated pattern of growth. This implies that the ventricles and the neurogenic niches are localized in the internal areas of the cerebral mass.

To identify the main neurogenic niches, we first performed immunofluorescence on sagittal (Figure S1) and horizontal (Figure S2) sections of *S. canicula* brains. We chose the proliferative cell nuclear antigen (PCNA) as a marker of proliferative activity and S100β as a marker for radial glial cells (RG) [28,48]. PCNA is expressed at its peak during G1/S phases of the cell cycle, and its expression decreases upon cell-cycle exit [49]. Thus, PCNA staining broadly identifies proliferative cells. As previously noted, the telencephalon of *S. canicula* is dominated by radial glia, with little presence of astroglia [50]. Some astrocyte-like cells are observed throughout the brain parenchyma, with evident association to the blood vessels as well (Figure S3), a feature previously observed in the developing olfactory bulb of *S. canicula* [51].

The staining pattern of PCNA and S100β along the rostro-caudal axis confirms the localization of the neurogenic niches around the brain ventricles, as described by Docampo-Seara et al. [38]. The labeling pattern on S100β+ cells clearly identified them as radial glia. In addition to the ventricular walls, we also found the presence of radial glial cells lining the outer border of the tissue in all brain tissue sections (examples shown in Figure S1e and Figure S2e). To confirm that PCNA immunoreactivity corresponds to the expression of *PCNA*, we cloned the cDNA of *pcna* from *S. canicula* and performed chromogenic in situ hybridization (ISH) to confirm the specificity of the immunostaining. We then decided to focus our analysis in five brain areas: the anterior telencephalon, posterior telencephalon, mesencephalon, anterior cerebellum, and cerebellum/rhombencephalon.

On coronal sections of these areas, we performed double immunofluorescence for various proteins in order to characterize the nature of the cells within this neurogenic niche.

To identify nonglial progenitors, we utilized the generic progenitor marker Mushasi-1 (Msi1), an RNA-binding protein necessary for neurogenesis and enriched in aNSC [52], which has already shown to have the same role in teleosts as well [53].

As already mentioned, PCNA is a marker that broadly stains cells with a proliferative potential, identifying cells both in the G1 and S phases. To identify actively dividing cells, we also used the marker phospho-Histone H3 (pH3), which shows its peak of expression in metaphase during mitosis, thus marking only actively dividing cells [54]. Given the extreme conservation of histone sequences, we consider this staining as specific.

To stain and identify newly differentiated neurons, we utilized an antibody against β-III-tubulin (β-IIITub) [55], while, to localize mature neurons outside of the neurogenic niche areas, we utilized an antibody against the neuronal nuclear protein (NeuN) [56,57].

Lastly, we identified in the neurogenic niches the presence of lysosomes containing aggregates of lipofuscin, an autofluorescent age marker, by realizing a costaining of S100β and the lysosomal-associated membrane protein 1 (Lamp1).

In the following paragraphs, we present our observations divided by anatomical areas.

### 2.1. Anterior Telencephalon

**Figure 1:**
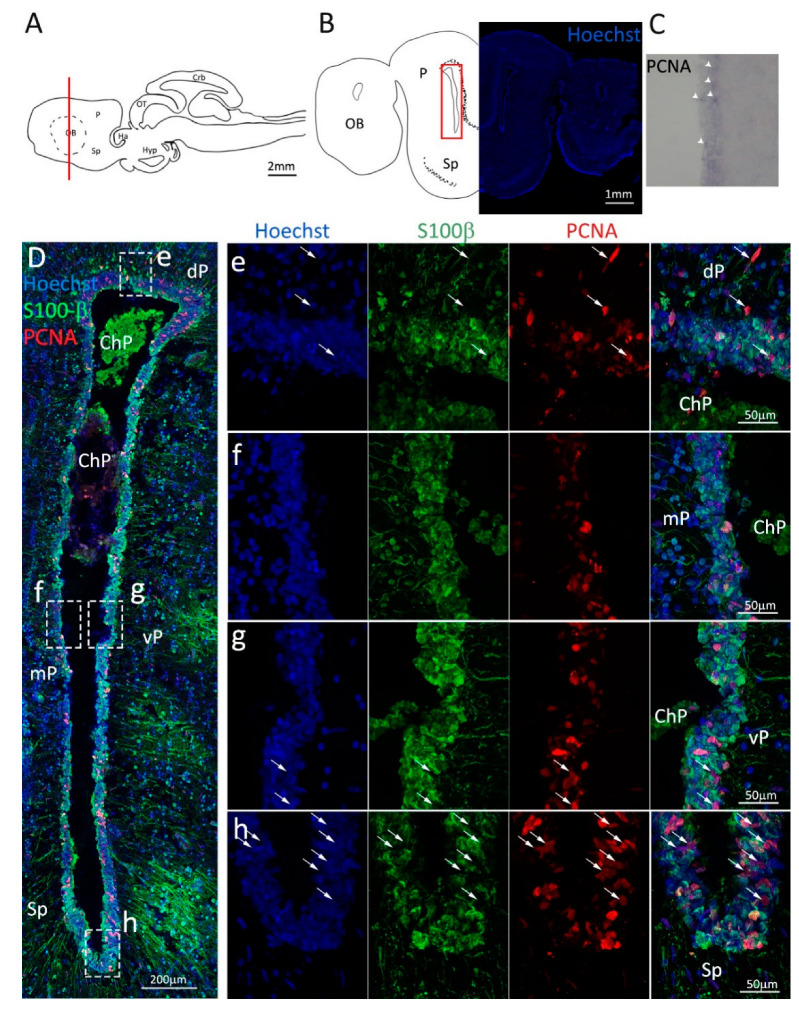
Localization of PCNA and S100β positive cells in the anterior telencephalon of *S. canicula*. (**A**) Sagittal representation of *S. canicula* brain. The red line indicates the rostro-caudal localization of the section represented in the panel. (**B**) Coronal map of the anterior telencephalon. On the right, a real coronal section is stained with Hoechst 33342 to show nuclei distribution; on the left, a cartoon is drawn to indicate anatomical references. Red rectangle identifies the general area from which images are taken. (**C**) *S. canicula* PCNA in situ hybridization in the anterior telencephalon ventricle. White arrowheads indicate cells expressing *pcna* mRNA. (**D**) Anterior telencephalic neurogenic niche. Radial glial cell bodies (S100β^+^, green) are localized around the ventricular wall and their processes spread into the surrounding tissue. Some S100β^+^ cells are also PCNA^+^ (red), indicating the proliferative state of the stem cells. (**e**) Magnification of the neurogenic niche localized in the dP. White arrows indicate PCNA^+^/S100β^−^ cells. (**f**) Magnification of the neurogenic niche localized in the mP. (**g**) Magnification of the neurogenic niche localized in the vP. White arrowheads indicate PCNA^+^/S100β^−^ cells. (**h**) Magnification of the neurogenic niche localized in the Sp. White arrowheads indicate PCNA^+^/S100β^−^ cells. Abbreviations: OB: olfactory bulb, P: pallium, Sp: subpallium, dP: dorsal pallium, mP: medial pallium, vP: ventral pallium, ChP: choroid plexus.

Firstly, we analyzed the coronal sections of *S. canicula* brains representing the anterior telencephalon (Figure 1A), where the ventricles are visible as paired bilateral structures (Figure 1B). The main part of the ventricle is localized in the pallium, while only its most ventral part is localized in the subpallium (Figure 1B). In situ hybridization for *S. canicula pcna* (Figure 1C) revealed that the expression of the mRNA is localized around the ventricle in the presumptive neurogenic niche area, and it is particularly expressed in isolated cells (Figure 1C, arrowheads). This staining is consistent with what is observed at the protein level by immunofluorescence (Figure 1D–h, red). The expression of PCNA is mainly localized in the neurogenic niche, as identified by the staining for S100β (Figure 1D–h, green). S100β clearly labels RG-shaped cells, recognizable by their radial processes and distributed all around the ventricle, with cell somas adjacent to the ventricle walls forming a relatively compact layer spanning 6–8 nuclei (Figure 1D). In the pallial area of the neurogenic niche (Figure 1e–g), the vast majority of PCNA^+^ cells are also S100β^+^, while in the subpallial region of the niche is notable the presence of an increased number of PCNA^+^/S100β^−^ cells (white arrowheads, Figure 1h). As previously described by Docampo-Seara et al. [38], we observed a higher number of PCNA^+^ cells in the region of the ventral and dorsal pallium (vP, dP, Figure 1e,g) as compared to the medial pallium (mP, Figure 1f). Interestingly, and in contrast to what was observed in the previous work cited above, we also observed a high number of PCNA^+^ cells in the subpallial area of the ventricle (Sp, Figure 1h).

**Figure 2:**
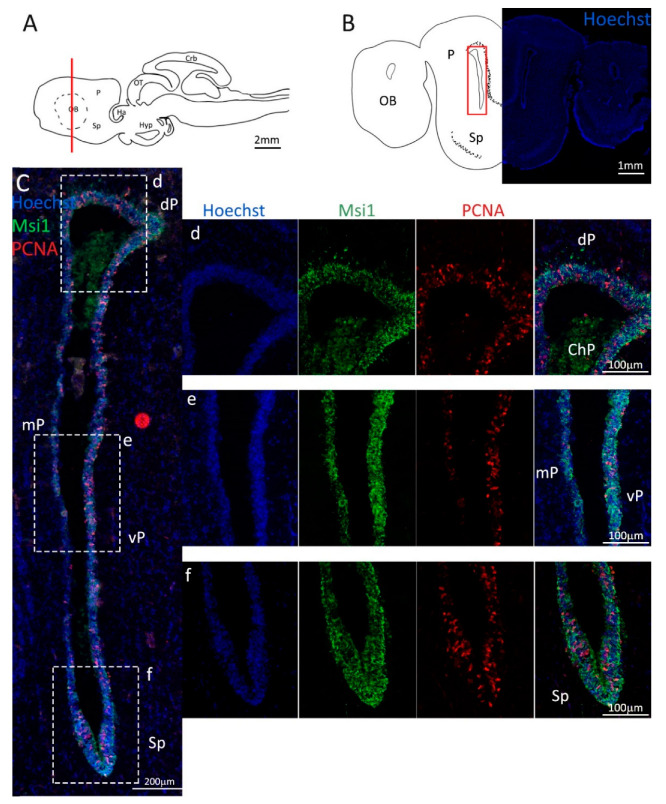
Localization of PCNA (red) and Msi1 (green) positive cells in the anterior telencephalon of *S. canicula*. (**A**) Sagittal representation of *S. canicula* brain. The red line indicates the rostro-caudal localization of the section represented in the panel. (**B**) Coronal map of the anterior telencephalon. On the right, a real coronal section is stained with Hoechst 33342 to show nuclei distribution; on the left, a cartoon is drawn to indicate anatomical references. Red rectangle identifies the general area from which images are taken. (**C**) Progenitor cells in the anterior telencephalic neurogenic niche. Progenitor cells (Msi1^+^, green) are localized around the ventricular wall. (**d**) Magnification of the neurogenic niche localized in the dP. (**e**) Magnification of the neurogenic niche localized in the mP and vP. It is evident the major thickness of the progenitor cells layer on the side of the vP in respect to the mP, as well as the higher number of PCNA^+^ cells (red). (**f**) Magnification of the neurogenic niche localized in the Sp. Abbreviations: OB: olfactory bulb, P: pallium, Sp: subpallium, dP: dorsal pallium, mP: medial pallium, vP: ventral pallium, ChP: choroid plexus.

Costaining of Msi1 and PCNA (Figure 2C–f, green and red, respectively) confirmed the localization of the progenitor cells tightly packed around the ventricle walls. On the vP side, the layer of progenitor cells appears thicker as compared to the area of the niche in the mV, and a higher number of proliferating cells are visible by the PCNA staining (Figure 2e). In fact, the progenitor cells localized in the mP appear to form the thinnest part of the niche, as compared to the dP, vP, and Sp areas (Figure 2d–f). It is of note that no Msi1^−^/PCNA^+^ cells are observed, an indication that the S100B^−^/PCNA^+^ cells observed, particularly in the Sp, are transient amplifying progenitors.

**Figure 3:**
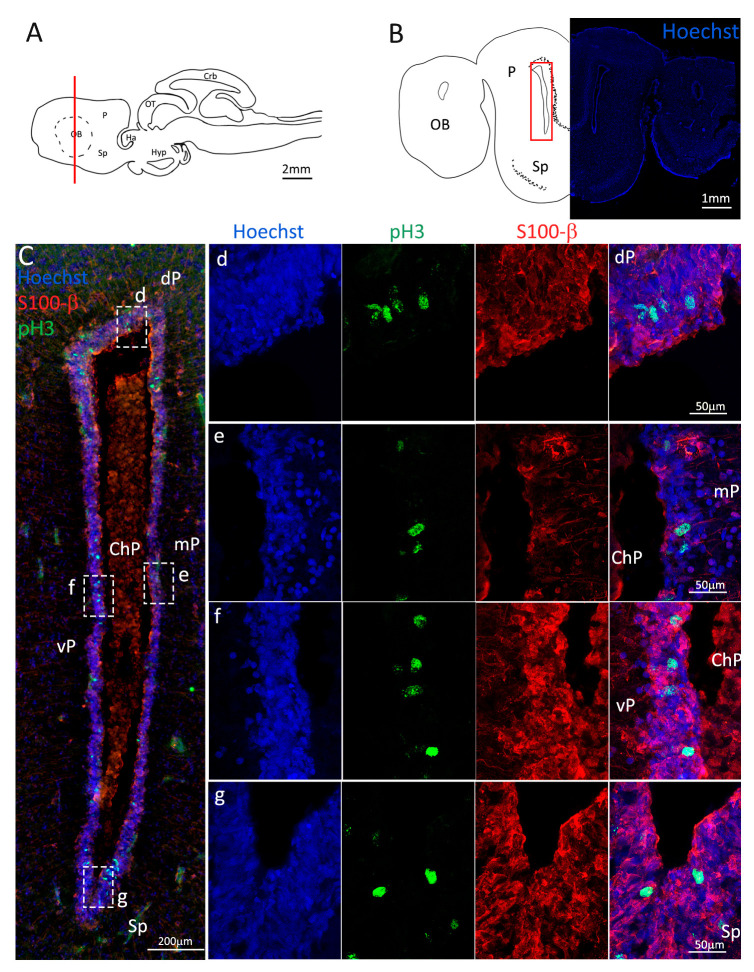
Localization of S100β (red) and pH3 (green) positive cells in the anterior telencephalon of *S. canicula*. (**A**) Sagittal representation of *S. canicula* brain. The red line indicates the rostro-caudal localization of the section represented in the panel. (**B**) Coronal map of the anterior telencephalon. On the right, a real coronal section is stained with Hoechst 33342 to show nuclei distribution; on the left, a cartoon is drawn to indicate anatomical references. Red rectangle identifies the general area from which images are taken. (**C**) Actively dividing cells (pH3^+^, green) in the anterior telencephalic neurogenic niche. (**d**) Magnification of the neurogenic niche localized in the dP. (**e**) Magnification of the neurogenic niche localized in the dP. (**f**) Magnification of the neurogenic niche localized in the mP. (**g**) Magnification of the neurogenic niche localized in the vP. Abbreviations: OB: olfactory bulb, P: pallium, Sp: subpallium, dP: dorsal pallium, mP: medial pallium, vP: ventral pallium, ChP: choroid plexus.

To confirm the presence of actively dividing cells, we performed a double staining for S100β and pH3 (Figure 3, red and green, respectively). We observed the presence of actively dividing cells in all areas of the niche (Figure 3C–g) and, also in this case, in the mP there are proliferative active cells (Figure 3e) but it appears that overall, the number of cells pH3^+^ is smaller than that in the areas of dP, vP and Sp (Figure 3C).

**Figure 4:**
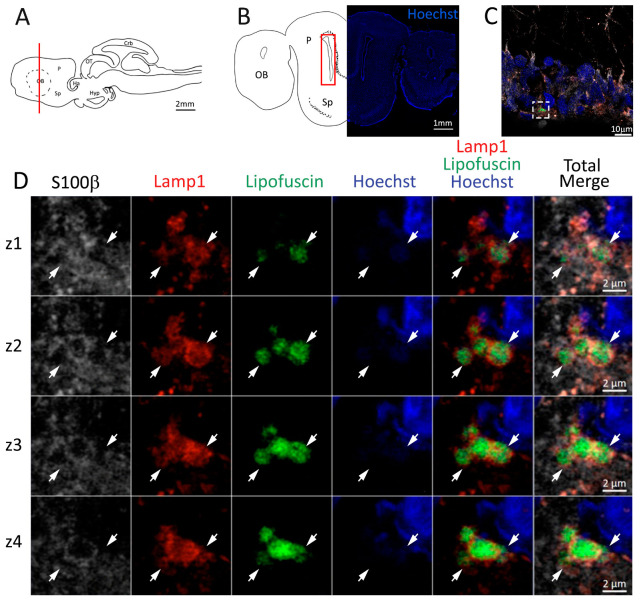
Presence of lipofuscin aggregates inside lysosomes in the anterior telencephalon of *S. canicula*. (**A**) Sagittal representation of *S. canicula* brain. The red line indicates the rostro-caudal localization of the section represented in the panel. (**B**) Coronal map of the anterior telencephalon. On the right, a real coronal section is stained with Hoechst 33342 to show nuclei distribution; on the left, a cartoon is drawn to indicate anatomical references. Red rectangle identifies the general area from which images are taken. (**C**) Area of the neurogenic niche where magnifications are taken from. (**D**) Lysosomes (Lamp1, red) containing lipofuscin (green) localized in radial glial cells (S100β^+^, white) and are localized around the nucleus (Hoechst, blue). In the panel, four consecutive z planes are represented (**z1**–**z4**) to render the three-dimensionality of the inclusion. Each plane is acquired at1 μm step in the z-axis from the previous. Last column represents the overlay of all four channels. White arrows indicate the localization of lysosomes containing lipofuscin. Abbreviations: OB: olfactory bulb, P: pallium, Sp: subpallium.

Utilizing an antibody against Lamp1, we were able to identify the lysosomes present in S100β^+^ cells of the neurogenic niche (Figure 4C). Many enlarged lysosomes were observed containing aggregates of the autofluorescent age marker lipofuscin (Figure 4C,D), as evident by the consequential imaging of multiple planes along the z axis in the confocal images (Figure 4D, white arrows). In the parenchyma outside the niche, enlarged lysosomes seem to be absent (Figure 4C), and we were not able to find any trace of lipofuscin autofluorescence in any other parenchyma region.

We also localized mature neurons, staining them with an anti-NeuN antibody (Figure S4C–E, green). As expected, the neurons are localized outside the periventricular neurogenic niche and no PCNA^+^/NeuN^+^ cells are detectable.

### 2.2. Posterior Telencephalon

**Figure 5:**
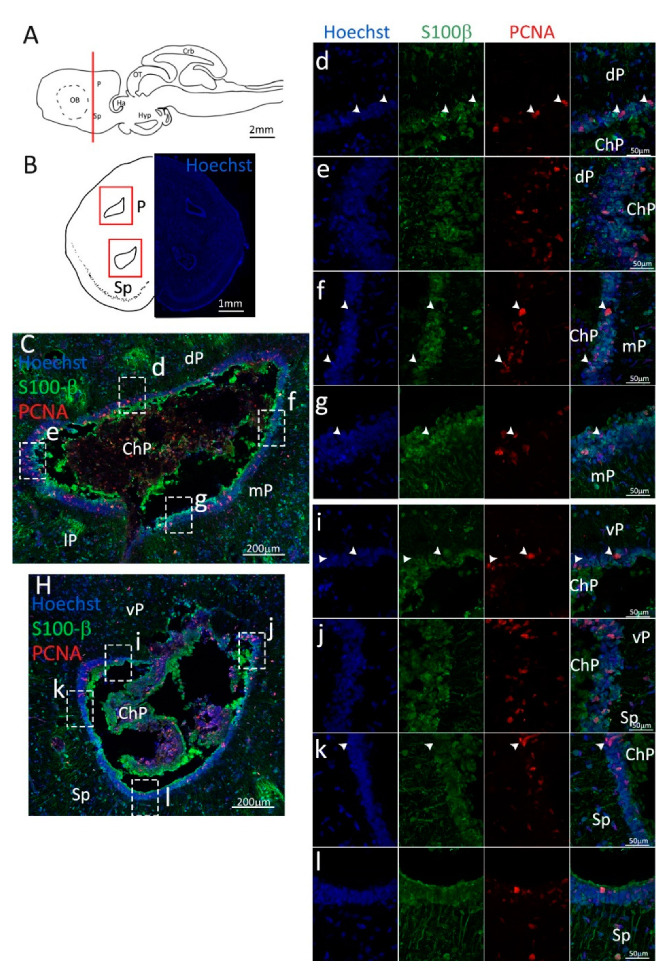
Localization of PCNA (red) and S100β (green) positive cells in the posterior telencephalon of *S. canicula*. (**A**) Sagittal representation of *S. canicula* brain. The red line indicates the rostro-caudal localization of the section represented in the panel. (**B**) Coronal map of the posterior telencephalon. On the right, a real coronal tissue section is stained with Hoechst 33342 to show nuclei distribution; on the left, a cartoon is drawn to indicate anatomical references. Red rectangles identify the general area from which images are taken. (**C**) Dorsal ventricle of the posterior telencephalon. This part of the ventricle is formed by dP, mP, and lP regions of the brain. Radial glial cells (S100β^+^, green) are tightly packed around the ventricle. (**d**) Magnification of the neurogenic niche localized in the dP. White arrowheads indicate PCNA^+^/S100β^−^ cells. (**e**) Magnification of the neurogenic niche localized in the dorsolateral pallium. (**f**) Magnification of the neurogenic niche localized in the mP. White arrowheads indicate PCNA^+^/S100β^−^ cells. (**g**) Magnification of the neurogenic niche localized in the mP. White arrowheads indicate PCNA^+^/S100β^−^ cells. (**H**) Ventral ventricle of the posterior telencephalon. This part of the ventricle is formed by vP and Sp regions of the brain. (**i**,**j**) Magnification of the neurogenic niche localized in the vP. White arrowheads indicate PCNA^+^/S100β^−^ cells. (**k**,**l**) Magnification of the neurogenic niche localized in the Sp. White arrowheads indicate PCNA^+^/S100β^−^ cells. Abbreviations: P: pallium, Sp: subpallium, dP: dorsal pallium, mP: medial pallium, lP: lateral pallium, vP: ventral pallium, ChP: choroid plexus.

In the posterior telencephalic area, the lateral ventricles collapse in the center and close tightly, resulting in the formation of two separate ventricles per side. One, more dorsal, is located entirely in the pallial area and formed by the dP, mP, and lP; the other is more ventral and located mainly in the Sp, with only its most dorsal region formed by the vP (Figure 5B). In this area, PCNA and S100β staining appeared similar to what was observed for the anterior telencephalon, resulting in a wall of S100β^+^ cells bodies lining the ventricle and radiating their neurites into the surrounding tissue (Figure 5C,H). In addition, in this case, the double-positive PCNA^+^/S100β^+^ cells appear to be less abundant in the mP region (Figure 5f,g) as compared to the dP and lP (Figure 5d,e). Differently from what is observed in the anterior telencephalon, the niche located in the Sp (Figure 5k,l) seems to have less proliferative active cells as compared to the part located in the vP (Figure 5i,j), as already described by other authors [38]. Another difference between anterior and posterior telencephalon consists of the presence of PCNA^+^/S100β^−^ cells: while anteriorly, we described an elevated number of these cells in the Sp niche, posteriorly, their number is greatly reduced (Figure 5k, white arrowheads).

**Figure 6:**
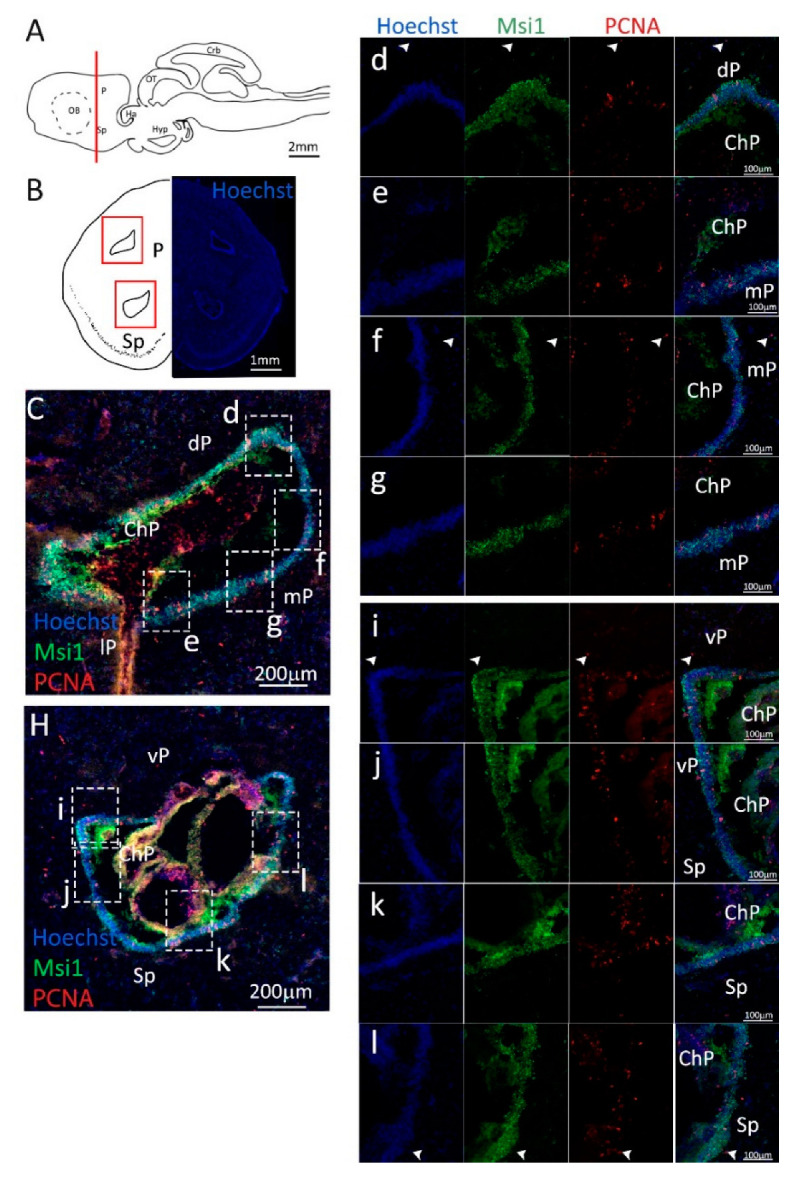
Localization of PCNA (red) and Msi1 (green) positive cells in the posterior telencephalon of *S. canicula*. (**A**) Sagittal representation of *S. canicula* brain. The red line indicates the rostro-caudal localization of the section represented in the panel. (**B**) Coronal map of the posterior telencephalon. On the right, a real coronal section is stained with Hoechst 33342 to show nuclei distribution; on the left, a cartoon is drawn to indicate anatomical references. Red rectangle identifies the general area from which images are taken. (**C**) Dorsal ventricle of the posterior telencephalon. This part of the ventricle is formed by dP, mP, and lP regions of the brain. Progenitor cells (Msi1^+^, green) are localized around the ventricular wall. (**d**) Magnification of the neurogenic niche localized in the dP. White arrowheads indicate PCNA^+^/Msi1^−^ cells. (**e**–**g**) Magnification of the neurogenic niche localized in the mP. White arrowheads indicate PCNA^+^/S100β^−^ cells. (**H**) Ventral ventricle of the posterior telencephalon. This part of the ventricle is formed by vP and Sp regions of the brain. (**i**,**j**) Magnification of the neurogenic niche localized in the vP. White arrowheads indicate PCNA^+^/Msi1^−^ cells. (**k**,**l**) Magnification of the neurogenic niche localized in the Sp. White arrowheads indicate PCNA^+^/S100β^−^ cells. Abbreviations: P: pallium, Sp: subpallium, dP: dorsal pallium, mP: medial pallium, lP: lateral pallium, vP: ventral pallium, ChP: choroid plexus.

Double-staining of Msi1 and PCNA (Figure 6, green and red, respectively) shows the presence of the expected conformation of packed progenitor cells around the ventricle (Figure 6C–l). Msi1 staining appears to be mainly cytoplasmic in *S. canicula*, and all PCNA^+^ cells in the niche are also Msi1^+^ (Figure 6d–l). Isolated PCNA^+^ cells are also present outside the tightly packed niche (Figure 6C,H). They appear to be mainly PCNA^+^/Msi1^−^ cells (Figure 6d,f,i,k, white arrowheads), whose nature remains unclear at this level of the investigation.

**Figure 7:**
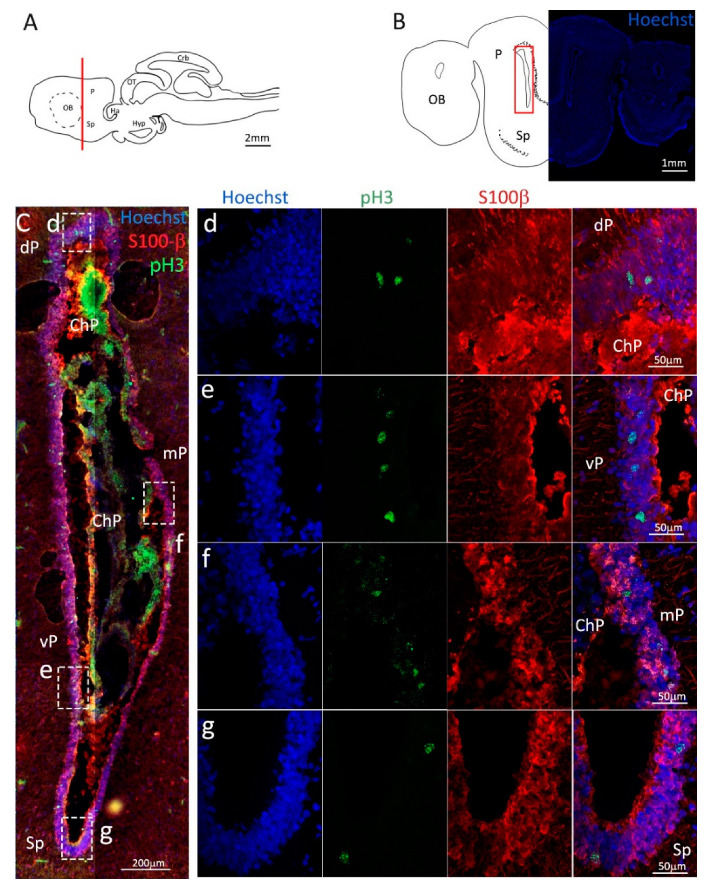
Localization of S100β (red) and pH3 (green) positive cells in the posterior telencephalon of *S. canicula*. (**A**) Sagittal representation of *S. canicula* brain. The red line indicates the rostro-caudal localization of the section represented in the panel. (**B**) Coronal map of the posterior telencephalon. On the right, a real coronal section is stained with Hoechst 33342 to show nuclei distribution; on the left, a cartoon is drawn to indicate anatomical references. Red rectangle identifies the general area from which images are taken. (**C**) Actively dividing cells (pH3^+^, green) in the posterior telencephalic neurogenic niche localize in the neurogenic niche (S100β^+^, red). (**d**) Magnification of the neurogenic niche localized in the dP. (**e**) Magnification of the neurogenic niche localized in the dP. (**f**) Magnification of the neurogenic niche localized in the mP. (**g**) Magnification of the neurogenic niche localized in the Sp. Abbreviations: OB: olfactory bulb, P: pallium, Sp: subpallium, dP: dorsal pallium, mP: medial pallium, vP: ventral pallium, ChP: choroid plexus.

Labeling with pH3 (Figure 7, green) revealed the presence of actively dividing cells in all pallial and subpallial areas, with the vP area showing a higher number of pH3^+^/S100β^+^ cells as compared to dP, mP, and Sp (Figure 7d–g).

**Figure 8:**
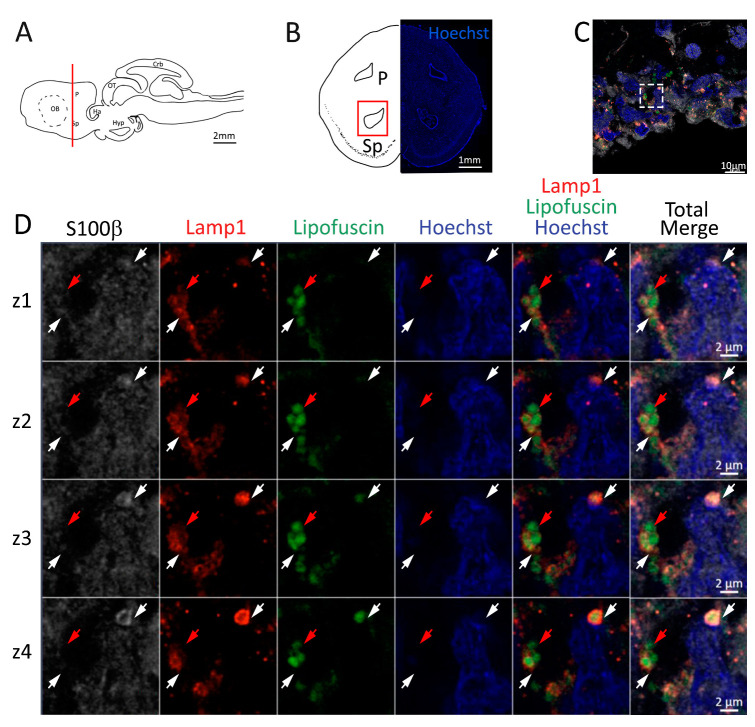
Presence of lipofuscin aggregates inside lysosomes in the posterior telencephalon of *S. canicula*. (**A**) Sagittal representation of *S. canicula* brain. The red line indicates the rostro-caudal localization of the section represented in the panel. (**B**) Coronal map of the posterior telencephalon. On the right, a real coronal section is stained with Hoechst 33342 to show nuclei distribution; on the left, a cartoon is drawn to indicate anatomical references. Red rectangle identifies the general area from which images are taken. (**C**) Area of the neurogenic niche where magnifications are taken from. (**D**) Lysosomes (Lamp1, red) containing lipofuscin (green) localized in radial glial cells (S100β^+^, white) and are localized around the nucleus (Hoechst, blue). In the panel, four consecutive z planes are represented (**z1**–**z4**) to render the three-dimensionality of the inclusion. Each plane is acquired at 1 μm step in the z-axis from the previous. Last column represents the overlay of all four channels. White arrows indicate the localization of lysosomes containing lipofuscin, red arrows indicate lipofuscin granules not included into lysosomes. Abbreviations: P: pallium, Sp: subpallium.

Lysosomes containing lipofuscin were also present in S100β^+^ cells in the posterior telencephalon (Figure 8C,D white arrows). Some lipofuscin granules appeared to be located in proximity of the lysosomes, but not enclosed by them (Figure 8D, red arrows).

Mature neuronal cells (NeuN^+^, Figure S5) remain located outside the neurogenic niche.

### 2.3. Mesodiencephalic Area

**Figure 9:**
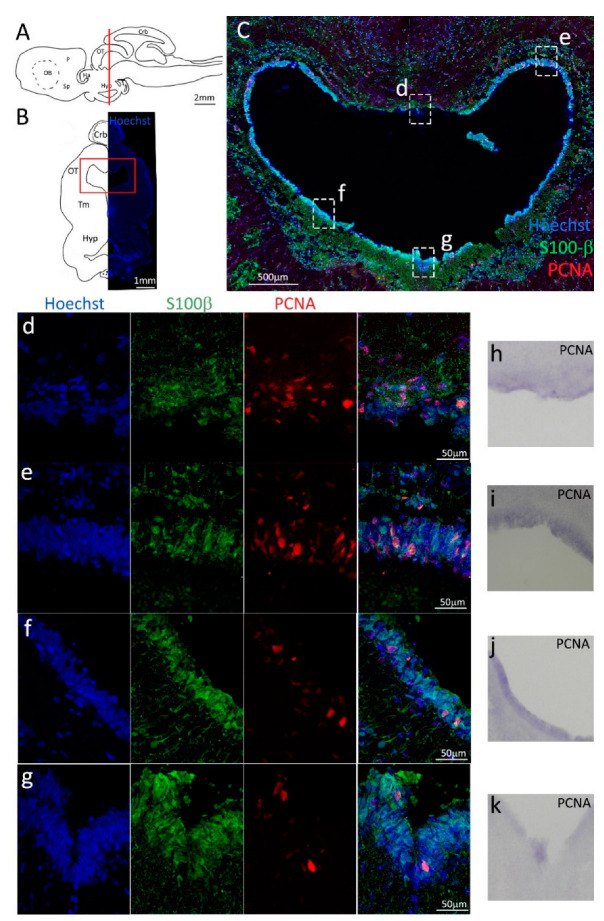
Localization of PCNA (red) and S100β (green) positive cells in the mesencephalon of *S. canicula*. (**A**) Sagittal representation of *S. canicula* brain. The red line indicates the rostro-caudal localization of the section represented in the panel. (**B**) Coronal map of the area imaged. On the right, a real coronal section is stained with Hoechst 33342 to show nuclei distribution; on the left, a cartoon is drawn to indicate anatomical references. Red rectangle identifies the general area from which images are taken. (**C**) Mesencephalic neurogenic niche. Radial glial cell bodies (S100β^+^, green) are localized around the ventricular wall and their processes spread into the surrounding tissue. Some S100β+ cells are also PCNA^+^ (red), indicating the proliferative state of the stem cells. (**d**,**e**) Magnification of the neurogenic niche localized in the tectal region. (**f**,**g**) Magnification of the neurogenic niche localized in the tegmental area. (**h**–**k**) In situ hybridization for *S. canicula pcna* in tectal and tegmental areas of the mesencephalic neurogenic niche. Abbreviations: Crb: cerebellum, OT: optic tectum, Tm: tegmentum, Hyp: hypothalamus.

The mesencephalic neurogenic niche is located around the heart-shaped mesencephalic ventricle (Figure 9A–C). Unlike what was observed in teleosts [58,59], the optic tectum of *Scyliorhinus canicula* shows no evident germinal layer that, in coronal sections, would appear as two crescents located on its medial and lateral margins. The dorsal part of the mesencephalic ventricular niche is located in the optic tectum (OT), while the most ventral part resides in the tegmental region (Tm, Figure 9B) [60]. S100β staining reveals a thinner layer of positive cells in the dorsal medial area of the niche of the optic tectum (Figure 9d, green), as compared to the dorsolateral part (Figure 9e, green). In the tegmental area, the thickness of the radial glial cell layer appears similar to that of the dorsolateral niche (Figure 9f,g, green). Despite the apparent thinner layer in its dorsomedial part, the tectal niche seems to arbor a higher density of PCNA^+^ cells (Figure 9d,e, red), if compared to the lateroventral and ventral portions of the ventricle located in the tegmentum (Figure 9f,g, red). PCNA in situ hybridization (Figure 9h–k) shows an mRNA localization more homogeneously distributed, resulting in a less-intense staining only in the most ventral part of the ventricle (Figure 9k).

**Figure 10:**
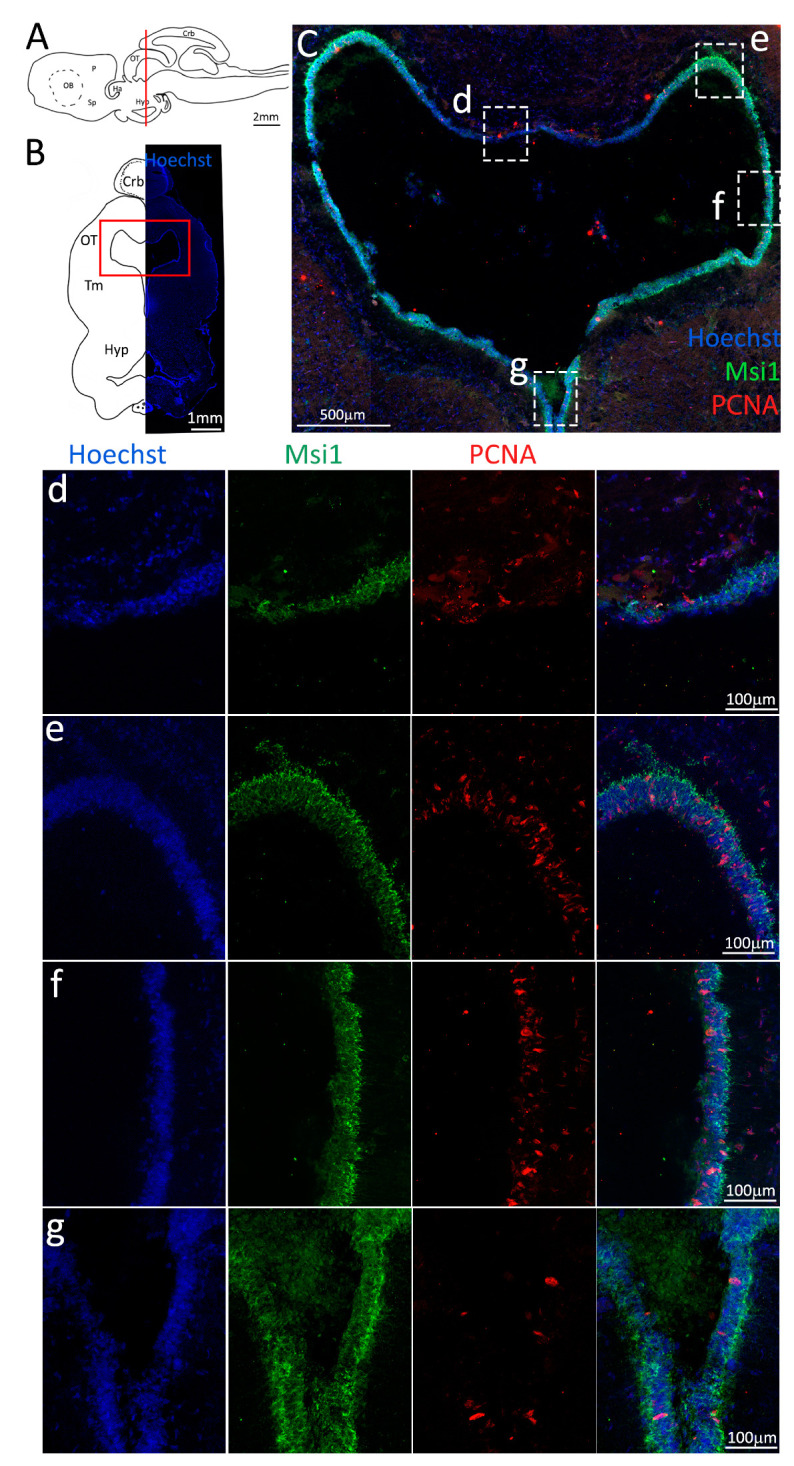
Localization of PCNA (red) and Msi1 (green) positive cells in the mesencephalon of *S. canicula*. (**A**) Sagittal representation of *S. canicula* brain. The red line indicates the rostro-caudal localization of the section represented in the panel. (**B**) Coronal map of the area imaged. On the right, a real coronal section is stained with Hoechst 33342 to show nuclei distribution; on the left, a cartoon is drawn to indicate anatomical references. Red rectangle identifies the general area from which images are taken. (**C**) Mesencephalic neurogenic niche. Progenitor cells (Msi1^+^, green) are localized around the ventricular wall. (**d**,**e**) Magnification of the neurogenic niche localized in the tectal region. (**f**,**g**) Magnification of the neurogenic niche localized in the tegmental area. Abbreviations: Crb: cerebellum, OT: optic tectum, Tm: tegmentum, Hyp: hypothalamus.

These observations were confirmed by the staining with Msi1 (Figure 10, green), showing a thinner layer of progenitor cells in the dorsomedial ventricle (Figure 10d) as opposed the other areas (Figure 10e–g).

**Figure 11:**
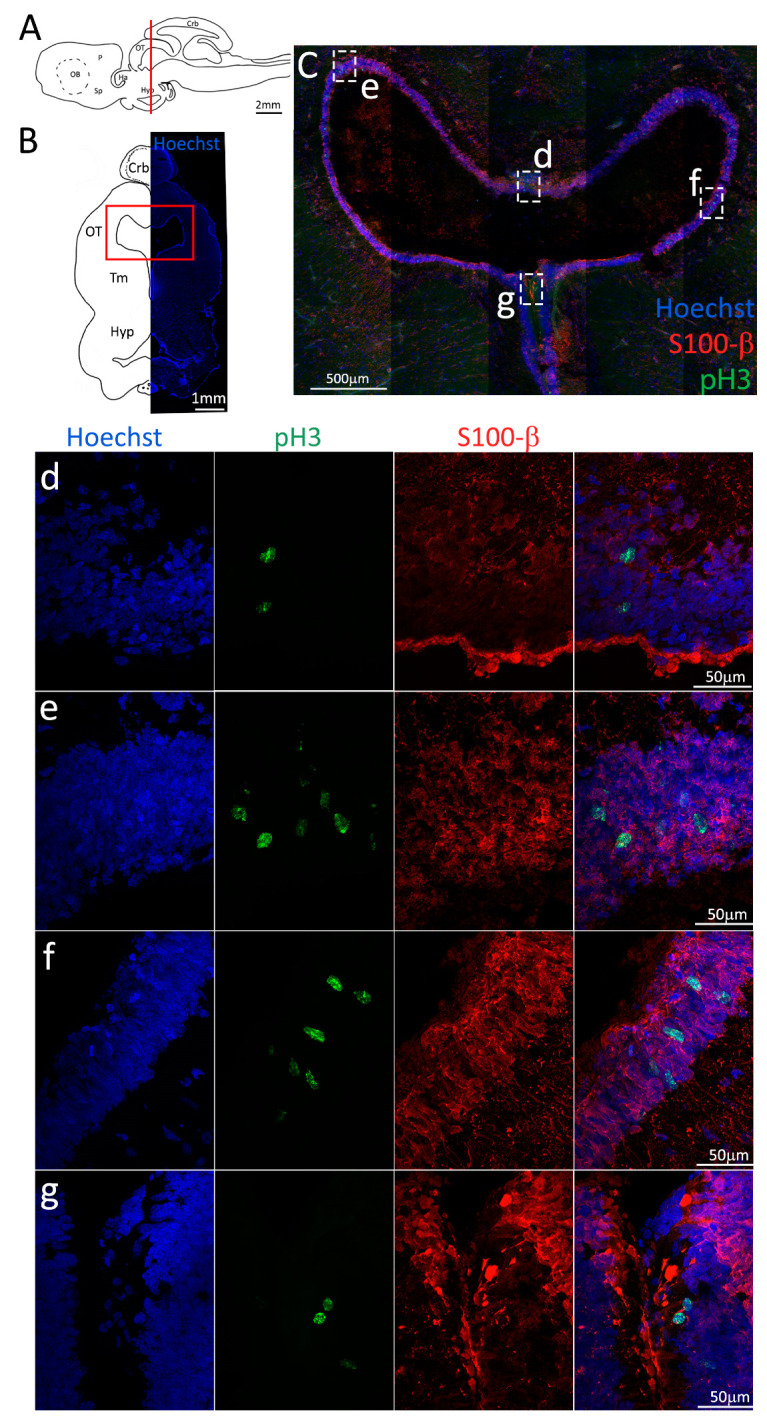
Localization of pH3 (green) and S100β (red) positive cells in the mesencephalon of *S. canicula*. (**A**) Sagittal representation of *S. canicula* brain. The red line indicates the rostro-caudal localization of the section represented in the panel. (**B**) Coronal map of the area imaged. On the right, a real coronal section is stained with Hoechst 33342 to show nuclei distribution; on the left, a cartoon is drawn to indicate anatomical references. Red rectangle identifies the general area from which images are taken. (**C**) Mesencephalic neurogenic niche. (**d**,**e**) Magnification of the neurogenic niche localized in the tectal region. (**f**,**g**) Magnification of the neurogenic niche localized in the tegmental area. Abbreviations: Crb: cerebellum, OT: optic tectum, Tm: tegmentum, Hyp: hypothalamus.

Somewhat surprisingly, we found a low amount of pH3^+^ cells in the dorsal side of the ventricle (Figure 11d), which is in contrast with the amount of PCNA staining observed in the same area (Figure 9d) and suggests the presence of slow-dividing cells. The number of dividing cells in other areas of the niche seems to reflect more closely the respective amount of PCNA observed, showing a higher number of pH3^+^ cells in the dorsolateral (Figure 11e) and lateral (Figure 11f) portions, as opposed to the ventral (Figure 11g) area.

**Figure 12:**
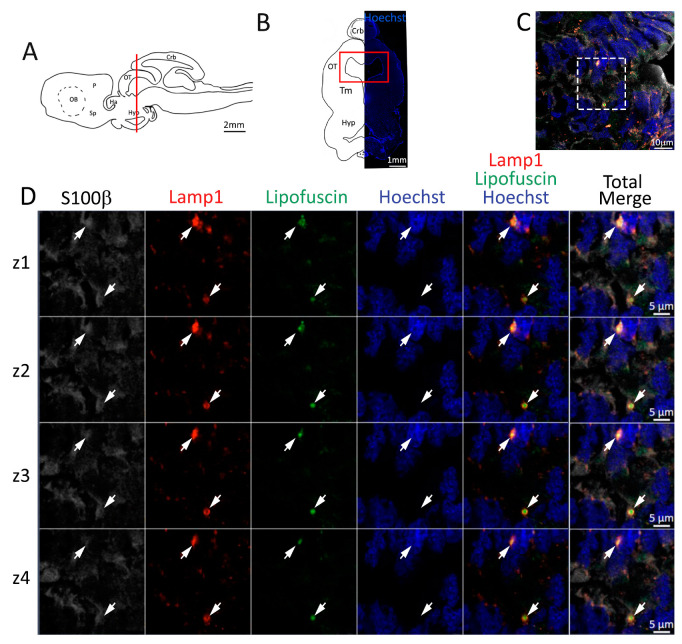
Presence of lipofuscin aggregates inside lysosomes in the mesencephalon of *S. canicula*. (**A**) Sagittal representation of *S. canicula* brain. The red line indicates the rostro-caudal localization of the section represented in the panel. (**B**) Coronal map of the area imaged. On the right, a real coronal section is stained with Hoechst 33342 to show nuclei distribution; on the left, a cartoon is drawn to indicate anatomical references. Red rectangle identifies the general area from which images are taken. (**C**) Area of the neurogenic niche where magnifications are taken from. (**D**) Lysosomes (Lamp1, red) containing lipofuscin (green) localized in radial glial cells (S100β^+^, white) and are localized around the nucleus (Hoechst, blue). In the panel, four consecutive z planes are represented (**z1**–**z4**) to render the three-dimensionality of the inclusion. Each plane is acquired at 1 μm step in the z-axis from the previous. Last column represents the overlay of all four channels. White arrows indicate the localization of lysosomes containing lipofuscin. Abbreviations: Crb: cerebellum, OT: optic tectum, Tm: tegmentum, Hyp: hypothalamus.

As already observed in the telencephalon, lipofuscin aggregates included in lysosomes are present in the radial glial cells of the mesencephalic niche (Figure 12). Mature neuronal cells (NeuN^+^, Figure S6) remain located outside the neurogenic niche, apart from some giant neuronal somata, which can be confidently classified as trigeminal motor neurons, similar to those observed in chickens [61,62].

Concerning the diencephalic niche, we observed the presence of PCNA^+^/S100β^−^ proliferating cells (Figure S7a,b, red), intercalated in a population of glial cells (S100β^+^, green) distributed homogeneously all around the ventricle. Few PCNA^+^/S100β^+^ cells can be detected, restricted to the dorsomedial area (Figure S7c).

### 2.4. Anterior Cerebellum

**Figure 13:**
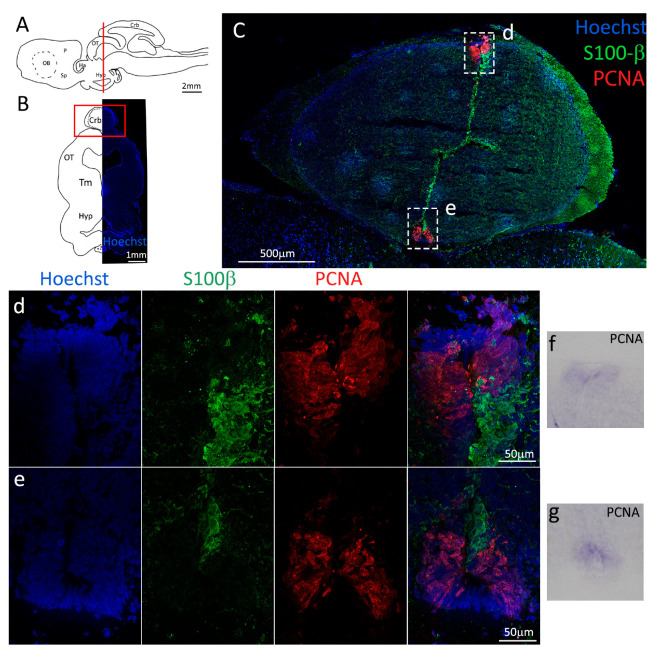
Localization of PCNA (red) and S100β (green) positive cells in the anterior cerebellum of *S. canicula*. (**A**) Sagittal representation of *S. canicula* brain. The red line indicates the rostro-caudal localization of the section represented in the panel. (**B**) Coronal map of the area imaged. On the right, a real coronal section is stained with Hoechst 33342 to show nuclei distribution; on the left, a cartoon is drawn to indicate anatomical references. Red rectangle identifies the general area from which images are taken. (**C**) Neurogenic niches of the anterior cerebellum. All PCNA^+^ cells (red) appear to be S100β^−^, suggesting a neuroepithelial origin of the stem cells. (**d**) Magnification of the neurogenic niche localized in the dorsal cerebellum. (**e**) Magnification of the neurogenic niche localized in the ventral cerebellum. (**f**,**g**) In situ hybridization for *S. canicula pcna* in dorsal and ventral cerebellar neurogenic niches. Abbreviations: Crb: cerebellum, OT: optic tectum, Tm: tegmentum, Hyp: hypothalamus.

Two main neurogenic niches are evident in the most anterior portion of the cerebellum (Figure 13A,B), as evidenced by S100β/PCNA immunostaining (Figure 13C) and *pcna* in situ hybridization (Figure 13f,g).

**Figure 14:**
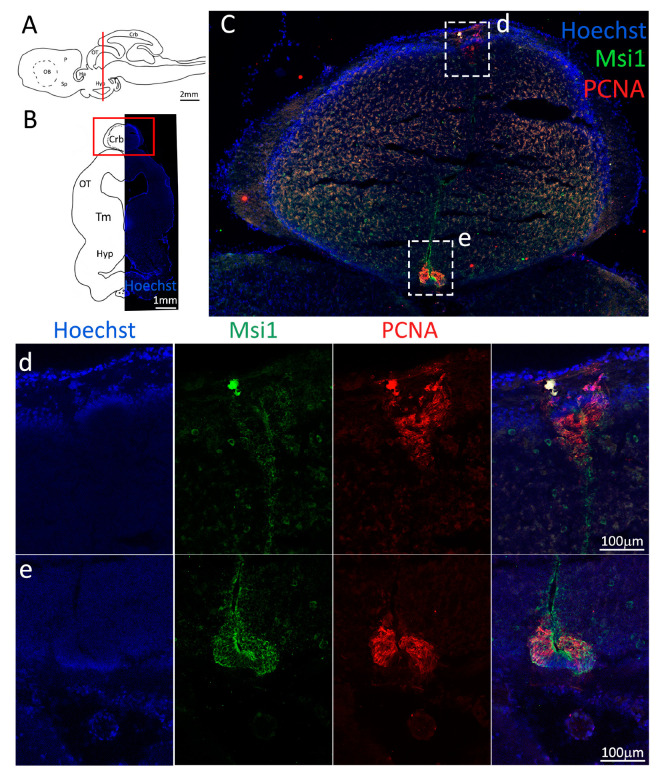
Localization of PCNA (red) and Msi1 (green) positive cells in the anterior cerebellum of *S. canicula*. (**A**) Sagittal representation of *S. canicula* brain. The red line indicates the rostro-caudal localization of the section represented in the panel. (**B**) Coronal map of the area imaged. On the right, a real coronal section is stained with Hoechst 33342 to show nuclei distribution; on the left, a cartoon is drawn to indicate anatomical references. Red rectangle identifies the general area from which images are taken. (**C**) Neurogenic niches of the anterior cerebellum. All PCNA^+^ cells (red) appear to be Msi1^+^, confirming their stem-cell nature. (**d**) Magnification of the neurogenic niche localized in the dorsal cerebellum. (**e**) Magnification of the neurogenic niche localized in the ventral cerebellum. Abbreviations: Crb: cerebellum, OT: optic tectum, Tm: tegmentum, Hyp: hypothalamus.

In this case, the cells composing the niches are not of radial glial nature but most likely of neuroepithelial origin: in fact, the niches are composed by a high number of proliferative active cells (PCNA^+^) of a typical columnar shape that show no costaining with S100β (Figure 13d,e), but express Msi1 (Figure 14C–e). It appears that most cells are in an active proliferative state (PCNA^+^/Msi1^+^), with only a few quiescent cells (PCNA^−^/Msi1^+^).

**Figure 15:**
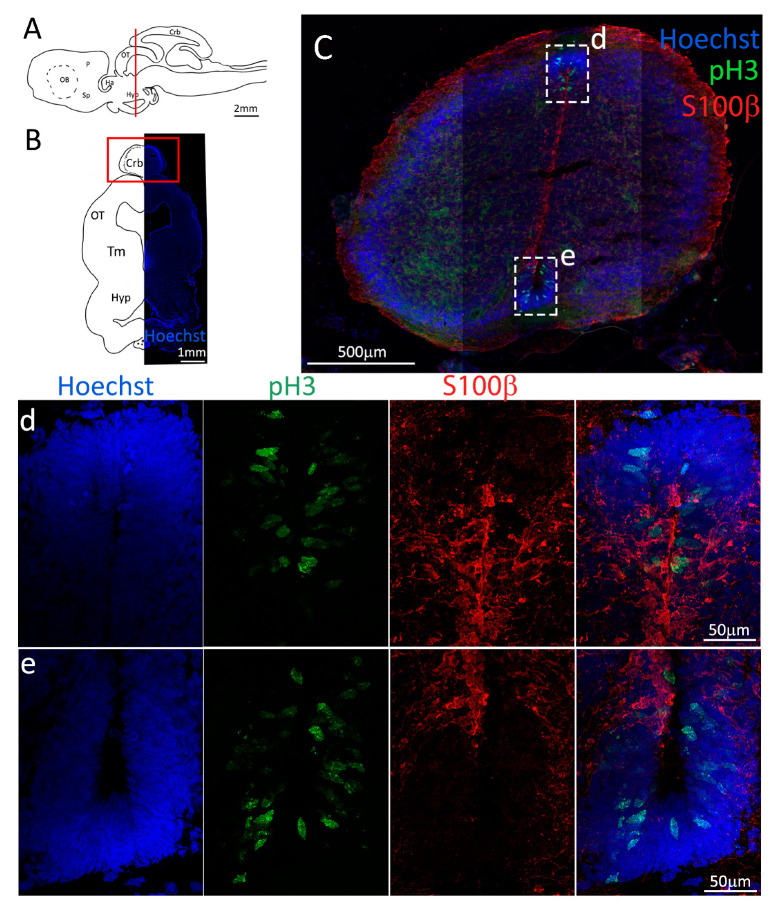
Localization of pH3 (green) and S100β (red) positive cells in the mesencephalon of *S. canicula*. (**A**) Sagittal representation of *S. canicula* brain. The red line indicates the rostro-caudal localization of the section represented in the panel. (**B**) Coronal map of the area imaged. On the right, a real coronal section is stained with Hoechst 33342 to show nuclei distribution; on the left, a cartoon is drawn to indicate anatomical references. Red rectangle identifies the general area from which images are taken. (**C**) Neurogenic niches of the anterior cerebellum. All pH3^+^ cells (red) appear to be S100β^−^. (**d**) Magnification of the neurogenic niche localized in the dorsal cerebellum. (**e**) Magnification of the neurogenic niche localized in the ventral cerebellum. Abbreviations: Crb: cerebellum, OT: optic tectum, Tm: tegmentum, Hyp: hypothalamus.

As expected, the number of pH3^+^ cells in the niches is lower than that of PCNA^+^ cells, confirming that progenitors in the niche are actively dividing (Figure 15C–e).

**Figure 16:**
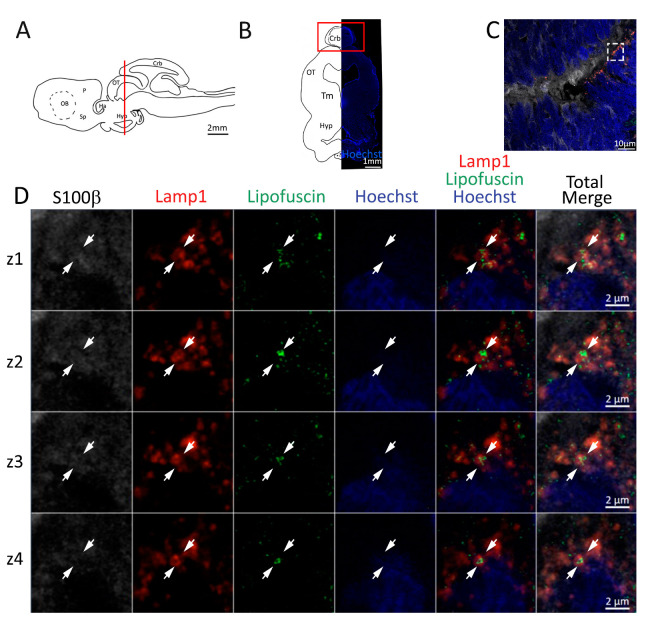
Presence of lipofuscin aggregates inside lysosomes in the anterior cerebellum of *S. canicula*. (**A**) Sagittal representation of *S. canicula* brain. The red line indicates the rostro-caudal localization of the section represented in the panel. (**B**) Coronal map of the area imaged. On the right, a real coronal section is stained with Hoechst 33342 to show nuclei distribution; on the left, a cartoon is drawn to indicate anatomical references. Red rectangle identifies the general area from which images are taken. (**C**) Area of the neurogenic niche where magnifications are taken from. (**D**) Lysosomes (Lamp1, red) containing lipofuscin aggregates (green). In the panel, four consecutive z planes are represented (**z1**–**z4**) to render the three-dimensionality of the inclusion. Each plane is acquired at 1 μm step in the z-axis from the previous. Last column represents the overlay of all four channels. White arrows indicate the localization of lysosomes containing lipofuscin. Abbreviations: Crb: cerebellum, OT: optic tectum, Tm: tegmentum, Hyp: hypothalamus.

In the anterior cerebellum, the general amount of lipofuscin seems lower than what is observed in other brain areas (Figure 16C), and few cells show signs of lysosomal staining containing lipofuscin aggregates (Figure 16D). Mature neuronal cells (NeuN^+^, Figure S8) remain located outside the neurogenic niche.

### 2.5. Cerebellum and Cerebellar Auricle

**Figure 17:**
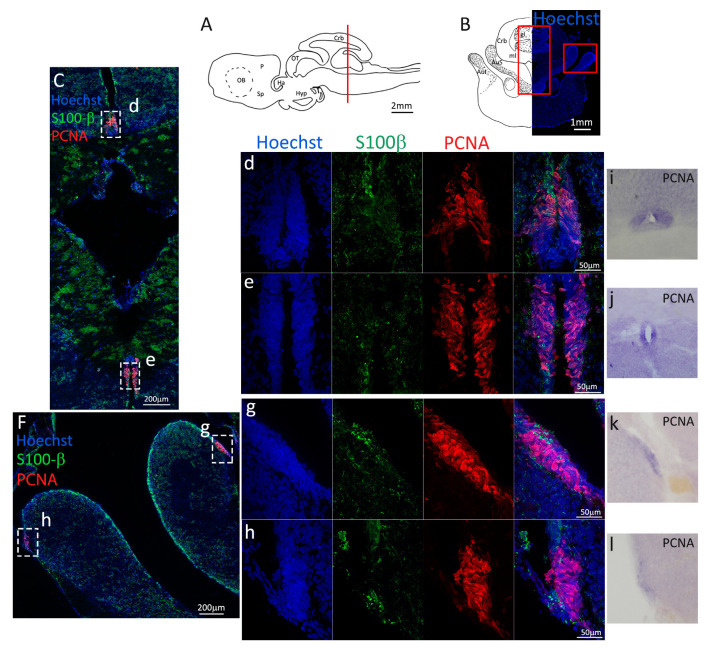
Localization of PCNA (red) and S100β (green) positive cells in the cerebellum of *S. canicula*. (**A**) Sagittal representation of *S. canicula* brain. The red line indicates the rostro-caudal localization of the section represented in the panel. (**B**) Coronal map of the area imaged. On the right, a real coronal section is stained with Hoechst 33342 to show nuclei distribution; on the left, a cartoon is drawn to indicate anatomical references. Red rectangles identify the general area from which images are taken. (**C**) Neurogenic niche of the cerebellum and medial dorsal auricle. (**d**) Magnification of the neurogenic niche localized in the ventral cerebellum. (**e**) Magnification of the neurogenic niche localized in the medial superior cerebellar auricle. (**F**) Lateral neurogenic niches of the cerebellar auricles. (**g**) Lateral neurogenic niche of the superior auricle. (**h**) Neurogenic niche of the inferior auricle. (**i**–**l**) In situ hybridization for *S. canicula pcna* in the cerebellum and cerebellar auricles. Abbreviations: Crb: cerebellum, gl: granular layer, ml: molecular layer, AuS: superior cerebellar auricle, AuI: inferior cerebellar auricle.

More posteriorly, the neurogenic niches are located at the interface between granular and molecular layers in the cerebellum and in the cerebellar auricles (Figure 17B). In the cerebellum, there is a dorsal (not shown) and a ventral niche (Figure 17C,d) positioned at the interface between the two layers. In the dorsal cerebellar auricle (AuS), two niches are identifiable: a medial one located in the molecular layer and adjacent to the granular layer (Figure 17C,e) and a dorsolateral one entirely positioned in the granular layer (Figure 17F,g). On the contrary, only one niche is identifiable in the inferior cerebellar auricle located in its granular layer (Figure 17F,h). As already described for the anterior cerebellar niches, the stem-cell features of both the cerebellum and cerebellar auricles suggest a neuroepithelial nature, lacking the S100β staining (Figure 17d–h) but showing a high number of PCNA^+^ cells, also supported by a diffuse staining of the corresponding areas via PCNA in situ hybridization (Figure 17i–l).

**Figure 18:**
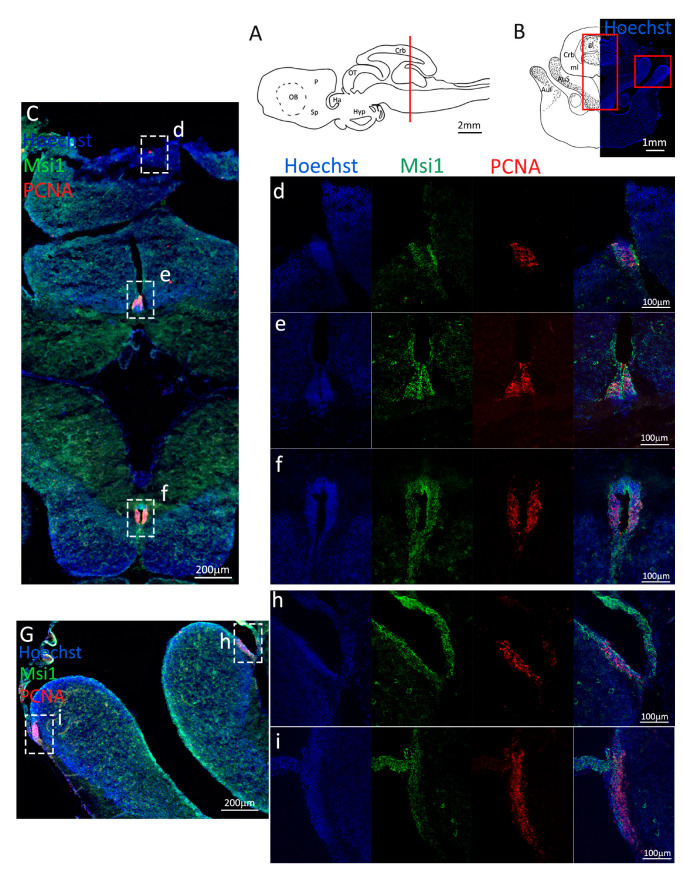
Localization of PCNA (red) and Msi1 (green) positive cells in the anterior cerebellum of *S. canicula*. (**A**) Sagittal representation of *S. canicula* brain. The red line indicates the rostro-caudal localization of the section represented in the panel. (**B**) Coronal map of the area imaged. On the right, a real coronal section is stained with Hoechst 33342 to show nuclei distribution; on the left, a cartoon is drawn to indicate anatomical references. Red rectangles identify the general area from which images are taken. (**C**) Neurogenic niche of the cerebellum and medial dorsal auricle. (**d**) Magnification of the neurogenic niche localized in the dorsal cerebellum. (**e**) Magnification of the neurogenic niche localized in the ventral cerebellum. (**f**) Magnification of the neurogenic niche localized in the medial superior cerebellar auricle. (**G**) Lateral neurogenic niches of the cerebellar auricles. (**h**) Lateral neurogenic niche of the superior auricle. (**i**) Neurogenic niche of the inferior auricle. Abbreviations: Crb: cerebellum, gl: granular layer, ml: molecular layer, AuS: superior cerebellar auricle, AuI: inferior cerebellar auricle.

Msi1 staining (Figure 18, green) confirmed the localization of the progenitor cells in the dorsal and ventral cerebellum (Figure 18C–e), as well as the two niches of the AuS (Figure 18C,f–h) and the only one of the AuI (Figure 18G,i).

**Figure 19:**
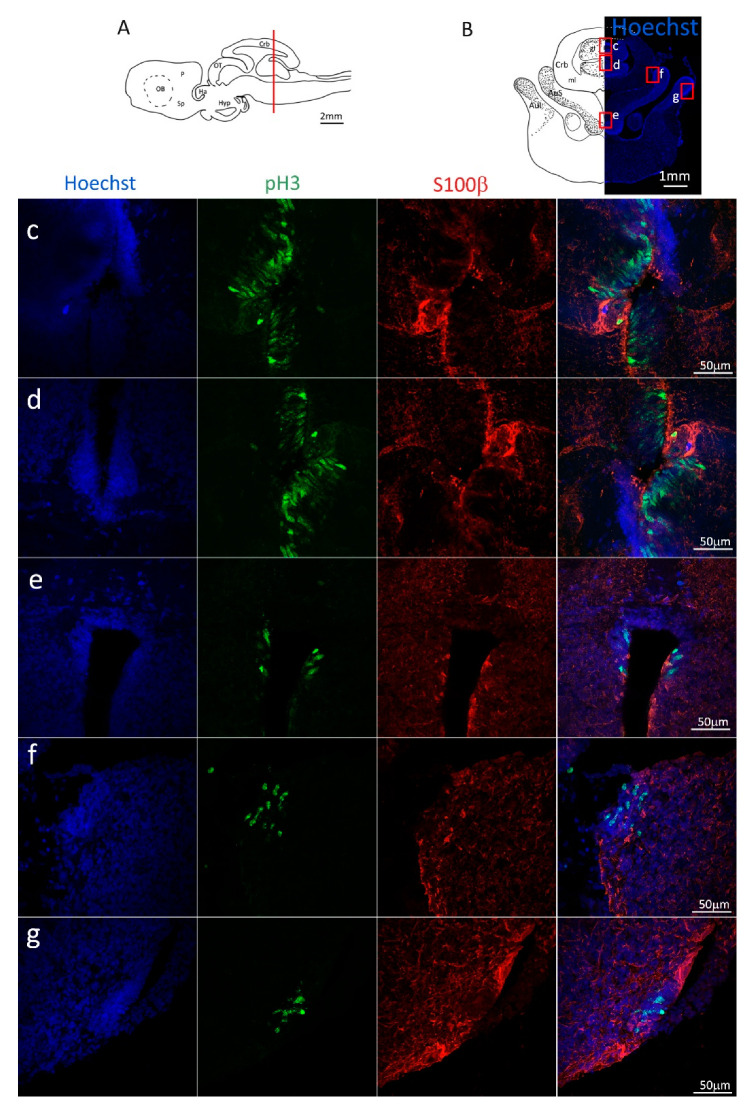
Localization of pH3 (green) and S100β (red) positive cells in the mesencephalon of *S. canicula*. (**A**) Sagittal representation of *S. canicula* brain. The red line indicates the rostro-caudal localization of the section represented in the panel. (**B**) Coronal map of the area imaged. On the right, a real coronal section is stained with Hoechst 33342 to show nuclei distribution; on the left, a cartoon is drawn to indicate anatomical references. Red rectangles identify the general area from which images are taken. (**c**) Magnification of the neurogenic niche localized in the dorsal cerebellum. (**d**) Magnification of the neurogenic niche localized in the ventral cerebellum. (**e**) Magnification of the neurogenic niche localized in the medial superior cerebellar auricle. (**f**) Lateral neurogenic niche of the superior auricle. (**g**) Neurogenic niche of the inferior auricle. Abbreviations: Crb: cerebellum, gl: granular layer, ml: molecular layer, AuS: superior cerebellar auricle, AuI: inferior cerebellar auricle.

pH3 staining (Figure 19, green) confirmed the presence of a really high number of actively dividing cells in the cerebellar niches (Figure 19c,d), and also a good number of dividing cells in the cerebellar auricles, even if to a lesser extent. The vast majority of pH3^+^ cells are S100β^−^, as expected.

**Figure 20:**
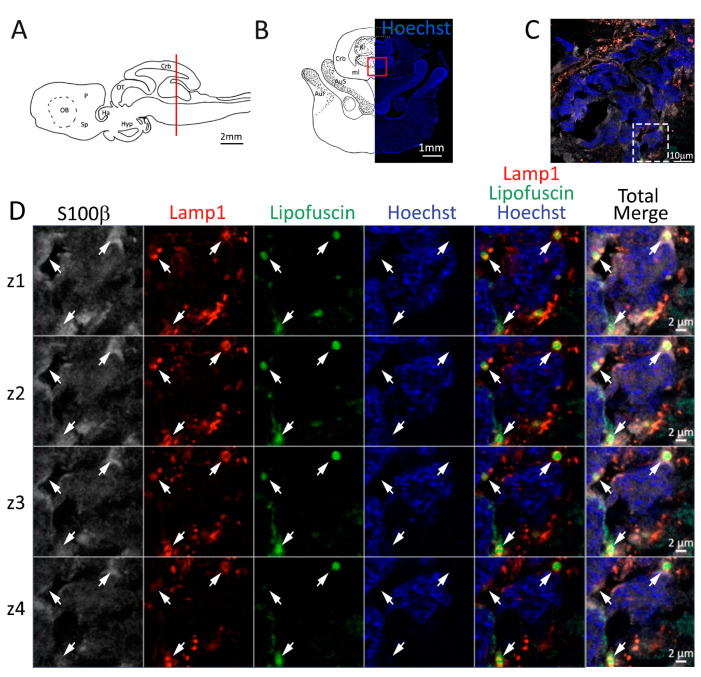
Presence of lipofuscin aggregates inside lysosomes in the cerebellum of *S. canicula*. (**A**) Sagittal representation of *S. canicula* brain. The red line indicates the rostro-caudal localization of the section represented in the panel. (**B**) Coronal map of the area imaged. On the right, a real coronal section is stained with Hoechst 33342 to show nuclei distribution; on the left, a cartoon is drawn to indicate anatomical references. Red rectangle identifies the general area from which images are taken. (**C**) Area of the neurogenic niche where magnifications are taken from. (**D**) Lysosomes (Lamp1, red) containing lipofuscin aggregates (green). In the panel, four consecutive z planes are represented (**z1**–**z4**) to render the three-dimensionality of the inclusion. Each plane is acquired at 1 μm step in the z-axis from the previous. Last column represents the overlay of all four channels. White arrows indicate the localization of lysosomes containing lipofuscin.

Presence of lysosomal staining containing lipofuscin aggregates (Figure 20) was found only in S100β^+^ cells localized around the most external cells of the niche (Figure 20C,D). Mature neuronal cells (NeuN^+^, Figure S9) remain located outside the neurogenic niche.

### 2.6. Effective Generation of New Neurons

**Figure 21:**
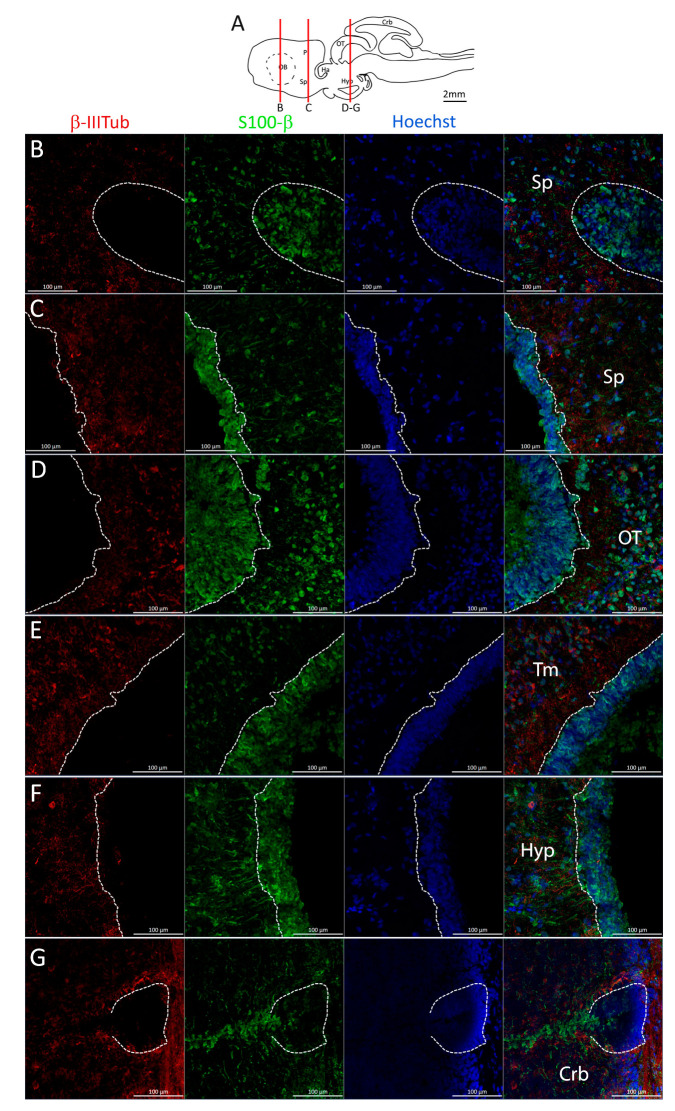
Identification of newly differentiated neurons in *Scyliorhinus canicula* brain. (**A**) Sagittal representation of *S. canicula* brain. The red lines indicate the rostro-caudal localization of the sections from which magnifications are taken. (**B**) Magnification of the neurogenic niche localized in the ventral area of the anterior telencephalon. (**C**) Magnification of the neurogenic niche localized in the ventral area of the posterior telencephalon. (**D**) Magnification of the neurogenic niche localized in the optic tectum. (**E**) Magnification of the neurogenic niche localized in the tegmentum. (**F**) Magnification of the neurogenic niche localized in the hypothalamus. (**G**) Magnification of the neurogenic niche localized in the dorsal anterior cerebellum. In all panels, white dashed lines indicate the border of the neurogenic niche. Abbreviations: Sp: subpallium, OT: optic tectum, Tm: tegmentum, Hyp: hypothalamus, Crb: cerebellum.

To assess the effective neurogenic activity of the identified niches, we performed a double-labeling with β-IIITub and S100β to specifically visualize newly differentiated neurons expressing β-IIITub (Figure 21, red). In all the anatomical areas analyzed, we observed the presence of new neurons localized just outside the neurogenic niche (Figure 21B–G). In the majority of the areas, β-IIITub staining was mutually exclusive with S100β (Figure 21B,D,E), but in the posterior telencephalon (Figure 21C), diencephalon (Figure 21F), and cerebellum (Figure 21G), we found presence of some labeling interspersed between the cells of the neurogenic niche on the parenchymal side.

## 3. Discussion

This paper represents the first systematic analysis of adult neurogenesis in the brain of the spotted catshark (*S. canicula*), extending the previous work which specifically analyzed the telencephalon.

Cartilaginous fishes represent the basal clade of gnathostomes and are, therefore, of particular interest to define the ancestral condition of adult neurogenesis. Adult neurogenesis has been studied in extreme detail in mammals, where it is restricted to the telencephalon and hypothalamus. Accordingly, the mammalian brain ceases to grow after childhood. Teleost fishes, on the other hand, show indefinite growth and extensive neurogenesis throughout the cerebral ventricles and, accordingly, the teleost brain keeps on growing in size throughout life. In addition, the teleost brain shows a heterochronic pattern, as some neural stem cells show a clear radial glial identity, whereas others retain a neuroepithelial phenotype. Another aspect of heterochrony is represented by the abundance of glial cells with radial morphology.

**Figure 22:**
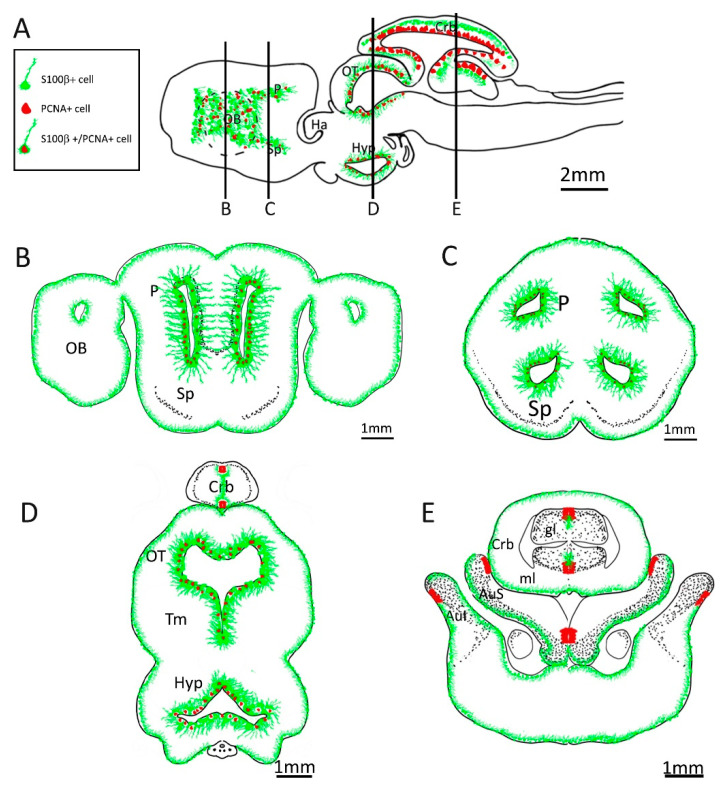
Schematic representation of adult neurogenic niches in *Scyliorhinus canicula* brain. (**A**) Representation of a sagittal section of *S. canicula* brain, with indicative localization of S100β^+^ (green) and PCNA^+^ (red) cells. (**B**) Representation of a coronal section of *S. canicula* anterior telencephalon, with indicative localization of S100β^+^ (green) and PCNA^+^ (red) cells around the ventricular walls. (**C**) Representation of a coronal section of *S. canicula* posterior telencephalon, with indicative localization of S100β^+^ (green) and PCNA^+^ (red) cells around the ventricular walls. (**D**) Representation of a mesodiencephalic coronal section of *S. canicula*, with indicative localization of S100β^+^ (green) and PCNA^+^ (red) cells. (**E**) Representation of a posterior coronal section of *S. canicula*, with indicative localization of S100β^+^ (green) and PCNA^+^ (red) cells in the cerebellum and cerebellar auricles. Abbreviations: OB: olfactory bulb, P: pallium, Sp: subpallium, Ha, habenulae, OT: optic tectum, Tm: tegmentum, Hyp: hypothalamus, Crb: cerebellum, gl: granular layer, ml: molecular layer, AuS: superior cerebellar auricle, AuI: inferior cerebellar auricle.

A comparison between teleost fishes and cartilaginous fishes, therefore, can shed some light on the putative phenotype of the gnathostomes ancestor. To characterize neuronal progenitors in catsharks, we used an antibody against S100β as a glial marker and an antibody against the RNA-binding protein Musashi-1 as pan-progenitor marker that labels glial cells, transit progenitors, and neuroepithelial progenitors. Here, we will discuss the different brain divisions separately (Figure 22).

### 3.1. Telencephalon

The telencephalon is divided into pallium and subpallium. In mammals, the subventricular zone is part of the subpallium and the subgranular zone is part of the pallium. Both niches contain stem cells of radial glia phenotypes and intermediate progenitors. The two niches differ clearly in their mitotic activity: the subpallial niche shows a higher neurogenic output that is sustained throughout adult life, and the hippocampal niche shows a much lower neurogenic output that declines rapidly during adult life. In teleosts, there are also clear differences between the two niches: the subpallial niche is highly active with fast-dividing cells as compared to the pallial niche. The stem cells in the subpallial niche do not express any glial markers, whereas neurogenic radial glia are present in the dorsal pallium. Interestingly, in the adult zebrafish, the dorsal pallium is completely tiled by radial glia, whilst nonglial progenitors persist in the dorsal pallium of the fast-growing turquoise killifish.

Our analysis is in line with the analysis of Docampo-Seara [38] in indicating that the lateral ventricles of the spotted catshark are lined by neurogenic radial glia. We did not find evidence of nonglial stem cells in the telencephalon of the spotted catshark, but our data are suggestive of the presence of intermediate progenitors that are placed near the radial glia and are particularly visible in the Sp. The neurogenic activity is not equally distributed along the different walls of the lateral ventricle, as already noticed by Docampo-Seara. In particular, the medial wall of the ventricle shows lower neurogenic activity. This situation is similar to that described in mammals where the septal wall is less neurogenic [63]. A comparison with mammals is, however, complicated by the different anatomical organization of the lateral ventricles. In mammals, the subventricular zone generates neurons of the olfactory bulb that migrate along the rostral migratory stream. In the spotted catshark, on the other hand, the lateral ventricle extends into the olfactory bulbs. During embryonic development, mitral cells are produced locally in the OB [39]; however, there is still the possibility that the pallial/subpallial neurogenic zones generate some neurons of the OB during adulthood.

### 3.2. Optic Tectum and Mesencephalon

The optic tectum is one of the most neurogenic regions of the teleost brain. The entire margin of the optic tectum is a germinal layer, where neuroepithelial cells ensure the circumferential growth of this structure [31,49,53,57,62]. The radial glia of the optic tectum is located in a thin layer that represents the most medial portion of the OT in direct contact with the ventricle. These cells show a very low neurogenic activity [53,63,64] and are characterized in teleosts by the expression of Msi1 [48,58]. In addition, in the optic tectum of the spotted catshark, we could detect a population of medially located radial glia, but we failed to detect a germinal zone so that the neurogenic activity in the OT seems to be sustained by Msi1^+^ radial glia located on the ventral surface. This result is consistent with the observation that a germinal layer (ciliary marginal zone) is present in juveniles of the spotted catshark, but its neurogenic activity becomes negligible in adults [45]. Neurogenic activity by radial glia is not restricted to the OT but is present along the entire dorsoventral extent of the third ventricle, with higher activity in the dorsolateral region.

### 3.3. Cerebellum

The cerebellum shows very active neurogenesis in teleost fishes [58,64,65,66]. The cerebellar niche is localized dorsally, maintaining contact with the ventricle, and is composed entirely of cells that retain a neuroepithelial phenotype [32]. The radial (Bergmann) glia of the cerebellum contributes neither to adult neurogenesis [32], nor to postinjury regeneration [67], but provides scaffolding of the niche [32]. Our results suggest that a very similar organization is present also in the spotted catshark. We clearly identified a circumscribed niche composed of tightly packed cells with a columnar organization that express Msi1 but no glial markers. A sharp border is observed between this niche and the layer or radial glia. As opposed to fishes, in the catshark we observed multiple niches, one for each subdivision of the cerebellum. In addition, similar niches were also observed in the cerebellar-like structures (auriculae) that are a specialized brain structure specific to cartilaginous fishes.

In summary, we can identify two important differences between teleost fishes and cartilaginous fishes: neither the telencephalon nor the optic tectum of cartilaginous fishes appear to contain stem cells of neuroepithelial origin. The neurogenesis in these two structures appear to be sustained entirely from stem cells of glial nature.

On the other hand, the role of neuroepithelial cells in cerebellar adult neurogenesis appears to be conserved between teleost fishes and cartilaginous fishes and, therefore, may represent a basal trait of gnathostomes.

### 3.4. Lipofuscin in Adult Neurogenesis

A surprising finding of our study is the presence of enlarged lysosomes containing lipofuscin aggregates in the neurogenic niches. These aggregates are observed exclusively in the S100β^+^ cells; they are not observed in neurons and are not observed in the neuroepithelial cells of the cerebellar neurogenic niche. Interestingly, lipofuscin granules are observed in the cerebellum in the radial glia in the close vicinity of the neuroepithelial niche. It is therefore likely that this specific accumulation of lipofuscin does not reflect an aging process, but it is specifically linked to the physiology of the radial glia.

In the rodent telencephalon, quiescent radial glial cells in the subventricular zone are characterized by the presence of enlarged lysosomes filled with aggregates that are strongly reminiscent of the enlarged lysosomes we described in the spotted catshark. Functional studies in rodents have revealed that quiescent radial glia shows upregulation of lysosomal genes and reduced activity of the proteasome. Activation of lysosomal activity increases the responsiveness of quiescent radial glial cells to mitogens [47].

Our results suggest that physiological accumulation of aggregates in quiescent radial glial cells is a conserved trait of gnathostomes.

### 3.5. Newly Generated Neurons

To demonstrate the effective generation of new neurons from the described neurogenic niches, we decided to perform double-labeling for β-III-tubulin and S100β in the same areas analyzed throughout the paper (telencephalon, diencephalon, mesencephalon, and cerebellum).

We decided to perform the staining with β-III-tubulin instead of the already-published staining with DCX [38] because, from in silico analysis, DCX seems to be not expressed in *S. canicula*. By running a BLASTp alignment of the human protein of DCX (NP_001182482.1) against all Sca annotated proteins, the best hit results in a protein annotated as DCX-like kinase 1 (DCLK1, XP_038673974.1, 94% query cover, 71% identities). By reverse-aligning this protein against the human database, the best hit results in human DCLK1 (XP_016876336.1, 99% query cover, 86% identities). Moreover, Docampo-Seara based their antibody specificity on the previous work of Pose-Mendez et al. [43], which utilized Western blot to prove specificity. However, the band observed is closer to the 49 kDa mark then 38 kDa. Considering that the molecular weight of DCX is 40 kDa and two of the isoforms of DCLK1 are 47 and 48 kDa, we assume that the Western blot is not conclusive in resolving the issue of DCX antibody specificity. Seeing all of the above, we concluded that the DCX stainings previously reported by the authors are likely due to cross-reaction of the DCX antibody with DCLK1.

On the other hand, by in silico comparing the human (NP_006077.2) and *S. canicula* β-III-tubulin (XP_038662401.1), the resulting alignment shows a query cover of 100% and a percentage of identity of 99%.

Staining for β-III-tubulin and S100β revealed an extensive presence of newly generated neurons outside of all the neurogenic niches identified in this work. In the majority of the areas, the staining appears to be mutually exclusive, indicating the presence of new neurons only outside the niche. However, in a few instances, some newly differentiated cells can be found along the parenchymal margin of the niche as well.

## 4. Materials and Methods

### 4.1. Tissue Collection and Preparation

Adult specimens of *Scyliorhinus canicula* were supplied alive by local fishermen. Following approbation of the Italian Ministry of Health (Cod. B290E.N.TU2), the animals were sacrificed. Brains were immediately dissected and fixed overnight in PFA 4%. The following day, the tissue was moved into a fresh solution of 30% sucrose for cryoprotection and left to equilibrate for 24–48 h at 4 °C. We then proceeded to embed the brain into OCT (Tissue-Tek O.C.T. Compound; Sakura Finetek, Torrance, CA, USA). Embedded brain tissue was snap-frozen in isopentane, then refrigerated at −80 °C until use. Serial sections of 45 μm were cut using a Leica cryostat and mounted on Superfrost Plus glass slides (Thermo Fisher Scientific, Waltham, MA, USA).

### 4.2. Total RNA Extraction, cDNA

Total RNA was extracted using the RNeasy Mini kit (Qiagen, Hilden, Germany) according to the manufacturer’s protocol and quantified using an FC-3100 (NanoReady, Steinfurt, Deutschland) spectrophotometer. Quality was checked by electrophoresis on agarose gel in RNAse-free conditions. An amount of 1 µg of total RNA was reverse-transcribed for cDNA synthesis with Reverse Transcriptase Core Kit (Eurogentec, Seraing, Belgium).

### 4.3. DIG-Labeled Riboprobe Synthesis

The template for *S. canicula pcna* was obtained by PCR amplification from cDNA using a forward (Fw: 5′-GCTCTACCGGCATCAGTTTG-3′) and a reverse primer carrying a T7 promoter sequence (underlined) on its 5′ end, (Re: 5′-TAATACGACTCACTATAGGGTGAAGAAGTTCAGGTACC-3′), designed on the *Sca pcna* (gene ID: 119957470). The PCR product was purified with Wizard^®^ SV Gel and PCR Clean-Up System (Promega, Madison, WI, USA) and verified via Sanger Sequencing (Eurofins Genomics, Louisville, KY, USA). Then, 50 ng were used as template to be directly transcribed with T7 RNA polymerase (Thermofisher Scientific, Waltham, NJ, USA) and digoxygenated RNTP mix (Roche, Basel, Switzerland) for 2 h at 37 °C. The resulting DIG-labeled riboprobe of 555 bp was precipitated with 1/10 of volume of LiCl (5 M) and 2.5 volume of ethanol ON at −20 °C, washed with 75% ethanol, resuspended in nuclease-free water, and stored at −80 °C. The quality and integrity of the riboprobe was assessed by gel electrophoresis performed in RNAse-free conditions.

### 4.4. Free-Floating In Situ Hybridization (ff-ISH)

ff-ISHs were performed according to [68], with some modifications. Sections (100 μm) were rehydrated in PBS, detached from the glass slice, and recovered in 2 mL safelock eppendorf (one section each). Sections were directly prehybridized for 30 min at 66 °C and then incubated with a digoxigenin DIG-labeled probe at 66 °C ON. Immediately before incubation, the probe was denatured at 80 °C for 3 min. Sections were washed twice for 15 min at 66 °C, firstly with SSC-2x and then with SSC-0.2x. Sections were treated with TMN solution (Tris-MgCl2-NaCl buffer) 3 times for 5 min, then stained with BM-Purple (Roche). The staining was constantly monitored under a stereomicroscope (M80 Leica, Wetzlar, Germany) equipped with an LED-light O-ring and blocked by washing in PBST. Once the color was fully developed, sections were postfixed in PFA 4% ON at 4 °C and coverslipped.

### 4.5. Double Immunofluorescence

To perform double immunofluorescence staining, we proceeded by rinsing the slides with PBS 1x, then performed acid antigen unmasking (citrate buffer, pH 6) for three minutes. The tissue was then blocked with a solution containing 5% BSA and 0.3% Triton-X 100 in PBS 1x for two hours at RT. Slides were then incubated overnight at 4 °C with a combination of primary antibodies, each at its proper dilution (Table 1). The following day, the slides were rinsed with PBS 1x three times and incubated, with the required combination of secondary antibodies diluted 1:500 for 2 h at RT. Sections were then rinsed again three times in PBS 1x, and nuclei were counterstained with Hoechst 33342 (Invitrogen, Waltham, MA, USA) and diluted 1:5000 in PBS 1x for one minute. The slides were then mounted with Fluoroshield mounting medium (Sigma, St. Louis, MO, USA). All incubations were performed in a humid chamber. The images of the negative control can be found in Supplementary Figure S10.

### 4.6. Immunofluorescence Using Monovalent Fab-Fragments Secondary Antibodies

To perform immunofluorescence utilizing two primary antibodies generated from the same host species, we utilized monovalent Fab fragments (AffiniPure Fab Fragment, Jakson ImmunoResearch, West Grove, PA, USA, Table 1). Monovalent Fab fragments are able to block immunoglobulins, allowing for staining utilizing same-host primary antibodies in two successive steps: the main difference with the standard double immunofluorescence consists of the separate and successive incubation of the two primary antibodies and respective secondary antibodies. Briefly, we proceed by incubating the tissue with the first primary antibody as described in the previous paragraph. The following day, the primary antibody is rinsed, and the monovalent Fab fragment conjugated with the proper fluorophore is applied at a 1:400 dilution. After 2 h at RT, the Fab fragment is rinsed three times in PBS 1x, and slides are then incubated with the second primary antibody ON at 4 °C. The following day, the second primary antibody is rinsed, and incubation with the required secondary antibody follows as described for the standard double immunofluorescence.

### 4.7. Imaging

Images of immunofluorescence-stained samples were acquired either with a Zeiss LSM900 Airyscan confocal microscope or with a Zeiss AxioScan microscope equipped with apotome slide. To realize the anatomical maps of the areas of interest, we imaged several tiles along the x–y axes utilizing a 10× objective with the Zeiss AxioScan. To obtain magnification of the single-niche areas, we acquired images with the confocal microscope with a 20× or 40× objective, acquiring 7 z-planes of each area in middle depth of the section thickness (approximatively in a range from 15 to 25 um of slice depth), each distanced 1 μm, and collapsing them into a maximum projection single image with the Zen suite. For each observed neurogenic region, we checked at least 450 um of surrounding brain tissue (10 slices) and included in the manuscript only a representative image of the area. ff-ISH whole-panoramic-view images were acquired with a Nikon Eclipse600 microscope equipped with DS-Fi3 color camera (Nikon, Tokyo, Japan) supplied with a double-LED-light O-ring. All images were adjusted for contrast and brightness using either the Zen Blue suite or Gimp. Panels were realized in Photoshop.

## 5. Conclusions

Summarizing from an evolutionary perspective, we can say that in addition to what has already been identified and described at the telencephalic level, there is also an interesting homology in neurogenic activity between chondrichthyans and mammals at the level of other regions, such as the hypothalamus and cerebellum. 

Regarding the first one, the existence of neurogenic activity within the mammalian hypothalamic area has been extensively described [69,70], and it has been associated to the regulation of metabolism, the circadian clock [71], and aging [72]. At the anatomical level, we have described in the hypothalamic area of *S. canicula* the presence of a component of radial glia, with morphology strongly resembling the tanycites that are well-described in the mammalian hypothalamic niches [73], and which are partially mitotically active.

Concerning the cerebellum, this is a very neurogenically active region in teleost fish [59], and we have described a strong cerebellar neurogenesis in chondrichthyans as well. On the other hand, the cerebellum is the last region of the brain that completes the developmental neurogenesis in mammals [74], which, in rodents, typically happens postnatally. It has been shown that even at the cerebellar level there are neurogenically active areas in some mammals, particularly in lagomorphs [75]. This creates a further parallel between the two groups.

Studying adult neurogenesis from a neuroethological and evolutionary perspective could provide a strategic approach to a deeper understanding of the phenomenon itself. As stated by Faykoo-Martinez and colleagues before us [76], and with whom we fully agree, “by stepping back and placing our findings in a much broader, non-biomedical context, we can help reduce dogmatic thinking and create a framework for discovery”.

## Figures and Tables

**Table 1:** List of all primary and secondary antibodies utilized in this work.

Primary Antibody	Producer	Catalog Number	Type	Working Dilution
Lamp-1	Abcam	ab24170	Polyclonal Rabbit	1:500
Msi-1	Cell Signaling	D46A8	Monoclonal Rabbit	1:100
NeuN	Abcam	Ab177487	Monoclonal Rabbit	1:500
PCNA	Dako	M0879	Monoclonal Mouse	1:500
pH3	Abcam	ab47297	Polyclonal Rabbit	1:250
β -IIITub	Abcam	ab78078	Monoclonal Mouse	1:2000
S100β	Genetex	GTX129573	Polyclonal Rabbit	1:500
**Secondary Antibody**				
AlexaFluor 488 anti-Rabbit	Invitrogen	A11001	Goat IgG	1:500
AlexaFluor 568 anti-Rabbit	Invitrogen	A11011	Goat IgG	1:500
AlexaFluor 568 anti-Mouse	Invitrogen	A11004	Goat IgG	1:500
AlexaFluor 635 anti-Rabbit	Invitrogen	A31576	Goat IgG	1:500
Alexa Fluor 488 AffiniPure Fab Fragment anti-Rabbit	Jakson ImmunoResearch	111547003	Goat/IgG (H+L)	1:400
Rhodamine Red-X (RRX) AffiniPure Fab Fragment anti-Rabbit	Jakson ImmunoResearch	111297003	Goat/IgG (H+L)	1:400

## Supplementary Materials

The following supporting information can be downloaded at: https://www.mdpi.com/article/10.3390/ijms24043650/s1.

## Abbreviations

AuS	Superior cerebellar auricle
AuI	Inferior cerebellar auricle
ChP	choroid plexus
Crb	cerebellum
dP	dorsal pallium
gl	granular layer
Ha	habenulae
Hyp	hypothalamus
lP	lateral pallium
ml	molecular layer
mP	medial pallium
OB	olfactory bulb
OT	optic tectum
P	pallium
Sp	subpallium
Tel	telencephalon
Tm	tegmentum
vP	ventral pallium
