# Supplementary material for: Localization and Characterization of Major Neurogenic Niches in the Brain of the Lesser-Spotted Dogfish Scyliorhinus canicula
Source: Int J Mol Sci. 2023 Feb 11;24(4):3650. doi: 10.3390/ijms24043650 (PMC9967623; doi:10.3390/ijms24043650)
Supplement: Supplementary file 1 [file ijms-24-03650-s001.zip › ijms-2181975-Supplementary.pdf]

Supplementary Materials

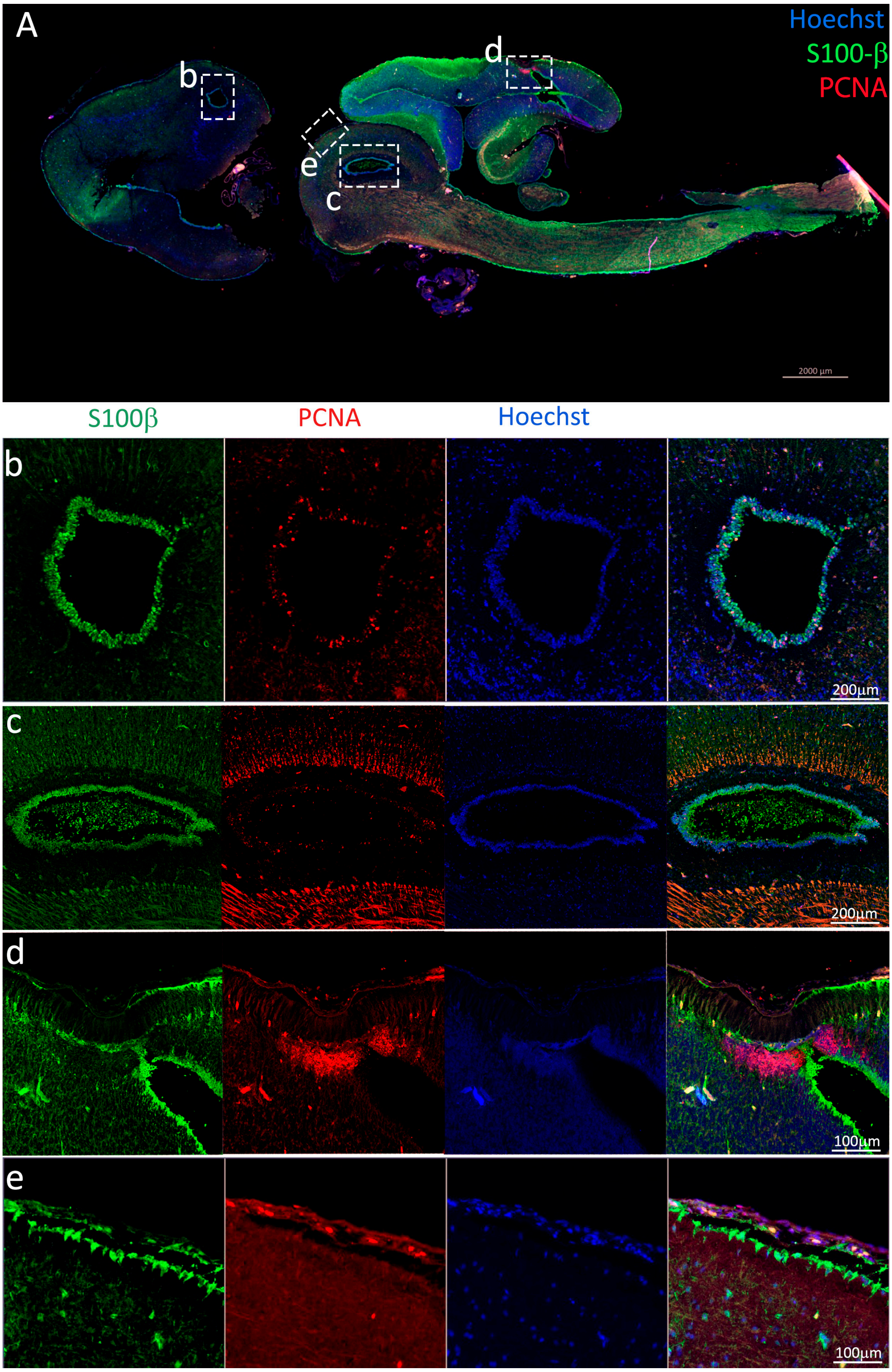

Figure S1. Localization of *S. canicula* neurogenic niches, sagittal. A) Sagittal section of *S. canicula* brain, stained for S100β (green) and PCNA (red). Dashed rectangles highlight magnified areas. b) Magnification of *S. canicula* telencephalic niche. c) Magnification of *S. canicula* mesencephalic niche. d) Magnification of *S. canicula* cerebellar niche. e) Example of radial glial cells (S100β+, green) localized at the external border of the section in the optic tectal area.

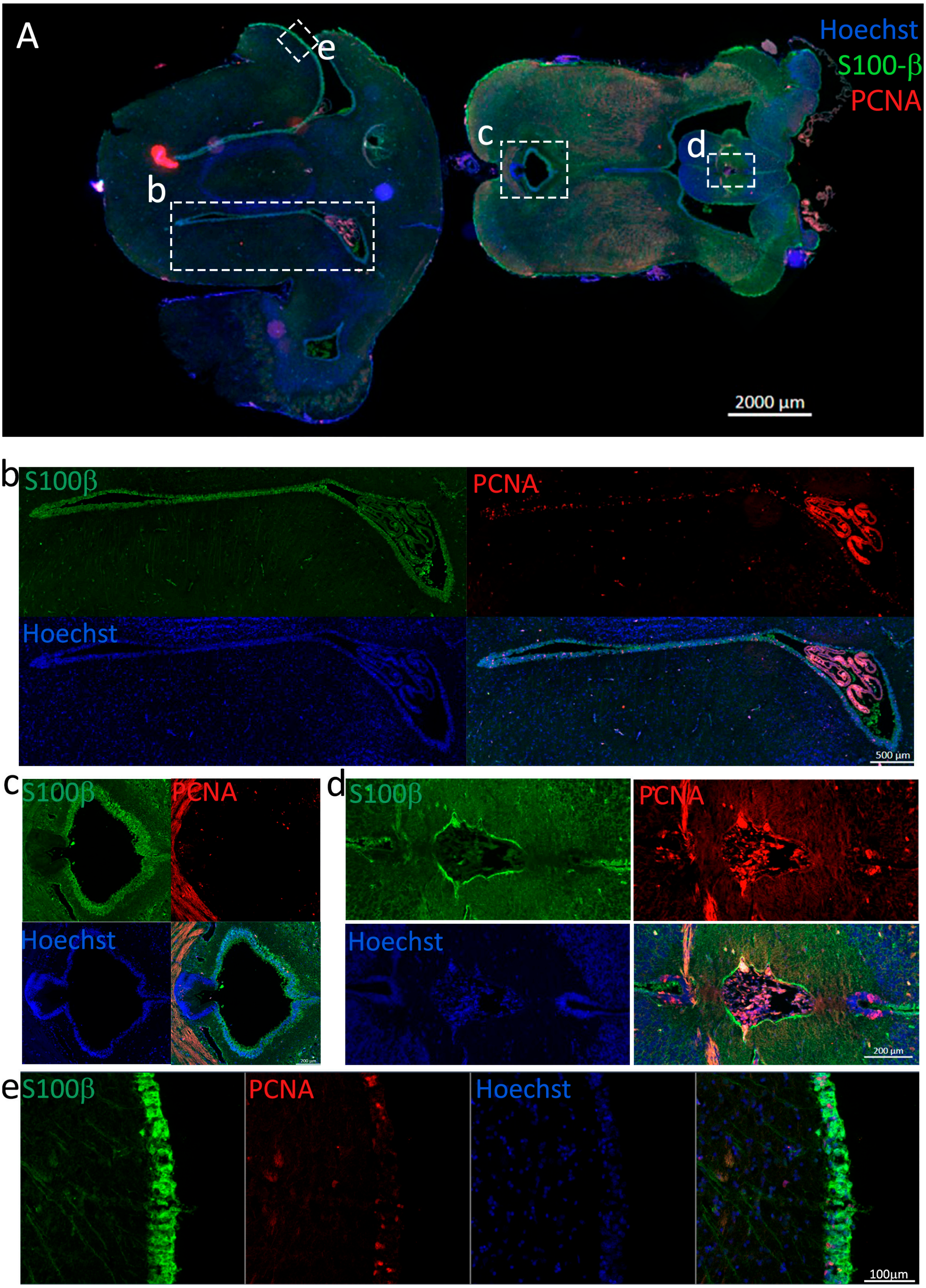

Figure S2. Localization of *S. canicula* neurogenic niches, horizontal. A) Horizontal section of *S. canicula* brain, stained for S100β (green) and PCNA (red). Dashed rectangles highlight magnified areas. b) Magnification of *S. canicula* telencephalic niche. c) Magnification of S. canicula mesencephalic niche. d) Magnification of *S. canicula* cerebellar niche. e) Example of radial glial cells (S100β^{+}, green) localized at the external border of the section in the telencephalic area.

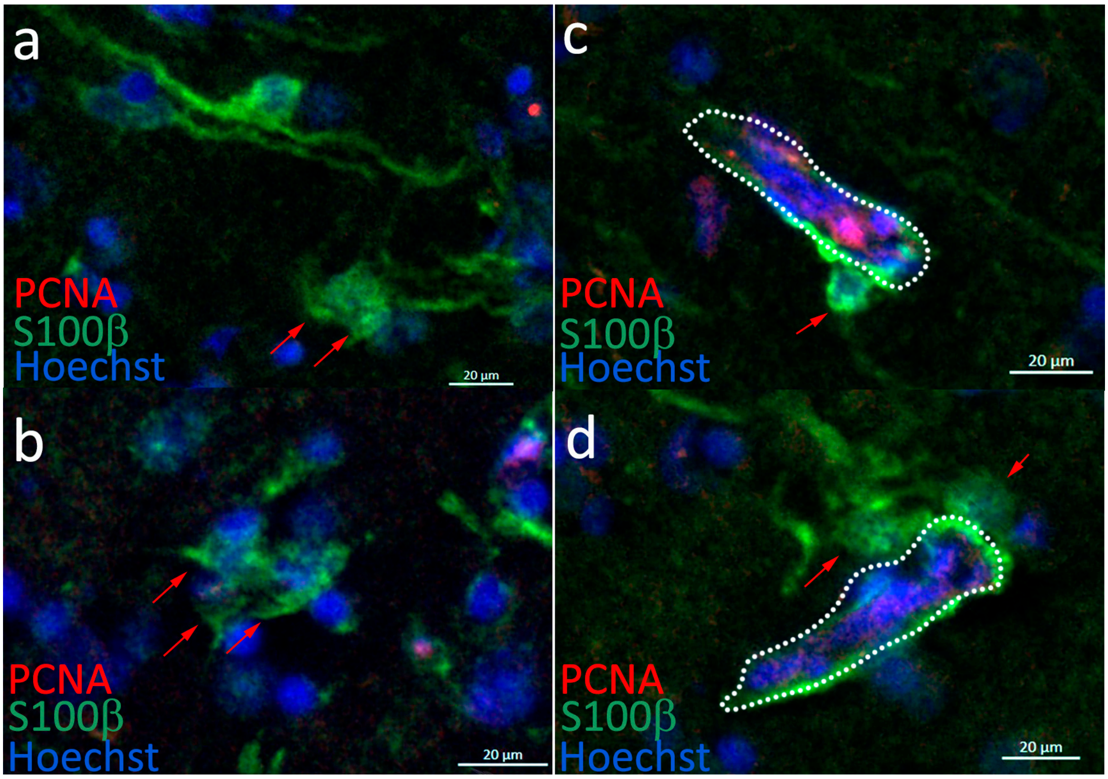

Figure S3. Presence of astroglial cells in the parenchyma of *S. canicula* brain. a-b) Astroglial cells (S100β$^+$, green), red arrows indicate astroglial cells. c-d) Astroglial cells (S100β$^+$, green) associated with blood vessels (autofluorescence, red). Dotted lines indicate blood vessel margins, red arrows indicate associated astroglial cells.

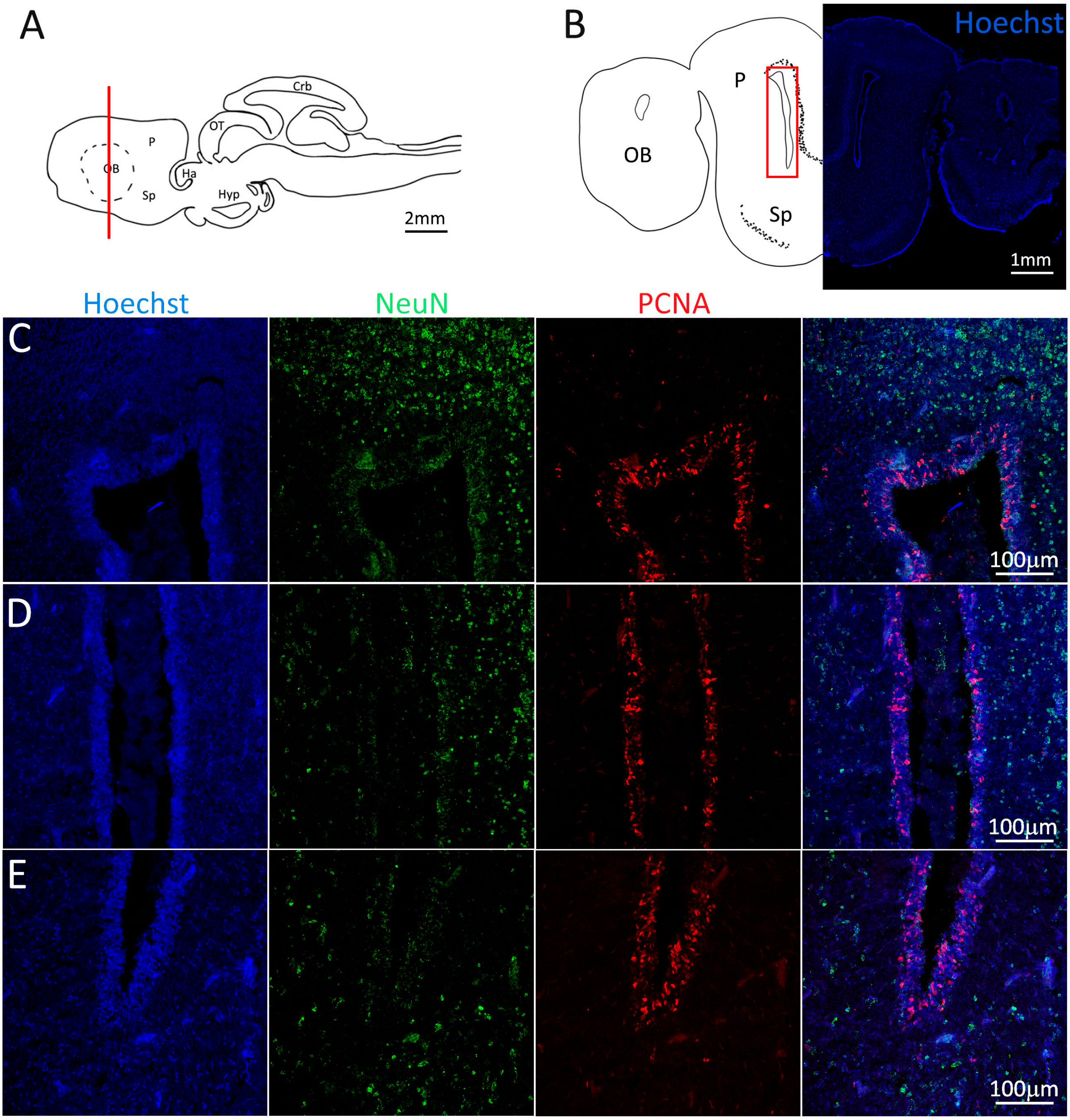

Figure S4. Localization of neuronal cells in the anterior telencephalon of *S. canicula*. A) Sagittal representation of *S. canicula* brain. The red line indicates the rostro-caudal localization of the section represented in the panel. B) Coronal map of the anterior telencephalon. On the right, a real coronal section is stained with Hoechst 33342 to show nuclei distribution, on the left a cartoon is drawn to indicate anatomical references. Red rectangle identifies the general area from which images are taken. C) Magnification of the neurogenic niche localized in the dP. Neuronal cells ($NeuN^+$, green) are located outside the neurogenic niche containing $PCNA^+$ cells (red). D) Magnification of the neurogenic niche localized in the mP and vP. Neuronal cells ($NeuN^+$, green) are located outside the neurogenic niche containing $PCNA^+$ cells (red) E) Magnification of the neurogenic niche localized in the Sp. Neuronal cells ($NeuN^+$, green) are located outside the neurogenic niche containing $PCNA^+$ cells (red).

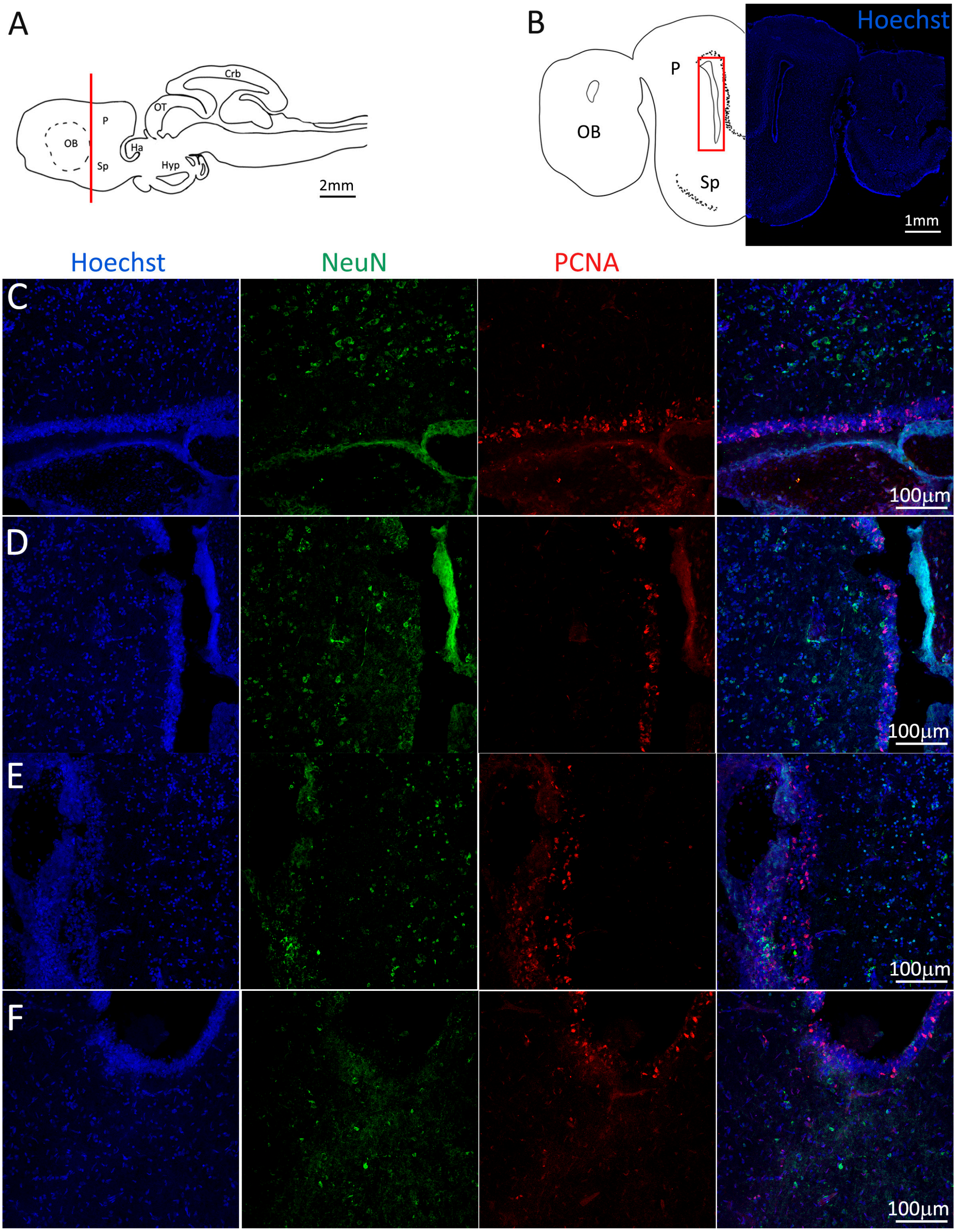

Figure S5. Localization of neuronal cells in the posterior telencephalon of *S. canicula*. A) Sagittal representation of *S. canicula* brain. The red line indicates the rostro-caudal localization of the section represented in the panel. B) Coronal map of the posterior telencephalon. On the right, a real coronal section is stained with Hoechst 33342 to show nuclei distribution, on the left a cartoon is drawn to indicate anatomical references. Red rectangle identifies the general area from which images are taken. C) Magnification of the neurogenic niche localized in the dP. Neuronal cells ($NeuN^+$, green) are located outside the neurogenic niche containing $PCNA^+$ cells (red). D) Magnification of the neurogenic niche localized in the mP. Neuronal cells ($NeuN^+$, green) are

located outside the neurogenic niche containing PCNA+ cells (red) E) Magnification of the neurogenic niche localized in the vP. Neuronal cells (NeuN+, green) are located outside the neurogenic niche containing PCNA+ cells (red). F) Magnification of the neurogenic niche localized in the Sp. Neuronal cells (NeuN+, green) are located outside the neurogenic niche containing PCNA+ cells (red).

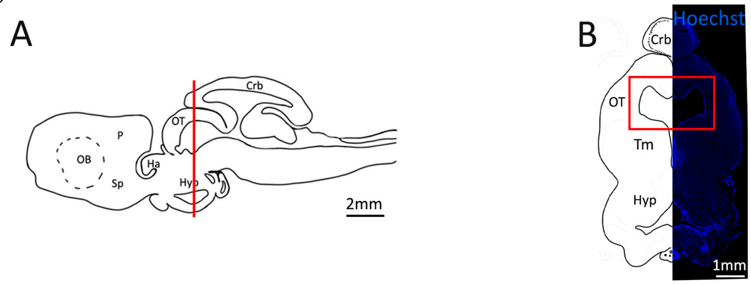

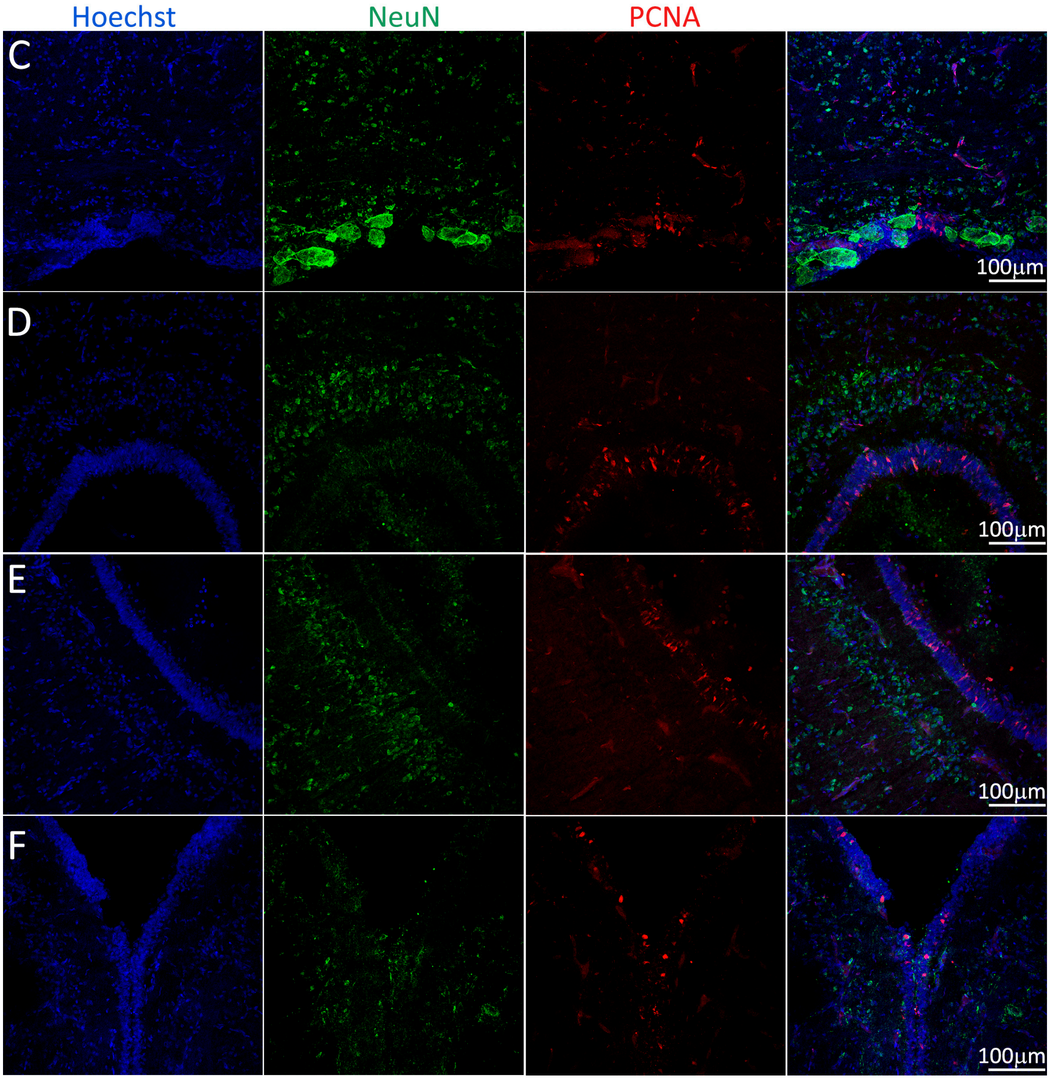

Figure S6. Localization of neuronal cells in the mesencephalon of *S. canicula*. A) Sagittal representation of *S. canicula* brain. The red line indicates the rostro-caudal localization of the section represented in the panel. B) Coronal map of the anterior telencephalon. On the right, a real coronal section is stained with Hoechst 33342 to show nuclei distribution, on the left a cartoon is drawn to indicate anatomical references. Red rectangle identifies the general area from which images are taken. C) Magnification of the neurogenic niche localized in the dorso-medial part of the optic tectum. The majority of neuronal cells (NeuN+, green) are located outside the neurogenic

niche containing PCNA⁺ cells (red). Giant trigeminal motor-neurons are interspersed within the cells of the neurogenic niche (NeuN⁺, green) D) Magnification of the neurogenic niche localized in the dorso-later portion of the niche located in the optic tectum. Neuronal cells (NeuN⁺, green) are located outside the neurogenic niche containing PCNA⁺ cells (red) E) Magnification of the neurogenic niche localized in the lateral part of the tegmentum. Neuronal cells (NeuN⁺, green) are located outside the neurogenic niche containing PCNA⁺ cells (red). F) Magnification of the neurogenic niche localized in the medial area of the tegmentum. Neuronal cells (NeuN⁺, green) are located outside the neurogenic niche containing PCNA⁺ cells (red).

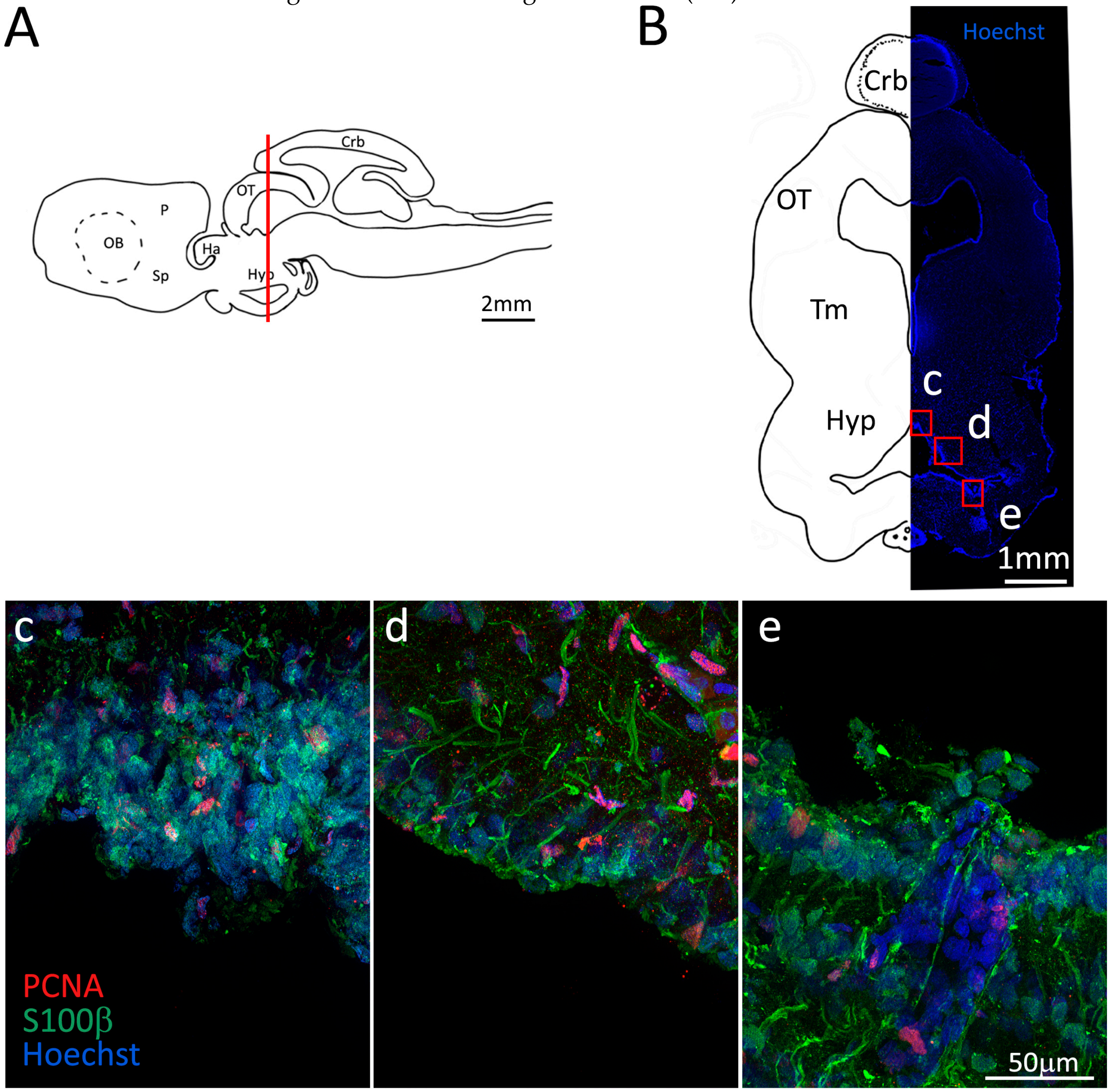

Figure S7. Localization of the adult neurogenic niche in the diencephalon of *S. canicula*. A) Sagittal representation of *S. canicula* brain. The red line indicates the rostro-caudal localization of the section represented in the panel. B) Coronal map of the area imaged. On the right, a real coronal section is stained with Hoechst 33342 to show nuclei distribution, on the left a cartoon is drawn to indicate anatomical references. Red rectangle identifies the general area from which images are taken. c) Magnification of the neurogenic niche localized in the dorso-medial part of the diencephalon. The majority of PCNA⁺ cells (red) are also S100β⁺ (green). d) Magnification of the

neurogenic niche localized in the dorso-later portion of the niche located in diencephalon. The majority of PCNA+ cells (red) appear to be S100β- (green). e) Magnification of the neurogenic niche localized in the ventro-lateral part of the diencephalon. The majority of PCNA+ cells (red) appear to be S100β- (green). The scale bar refers to panels c, d and e.

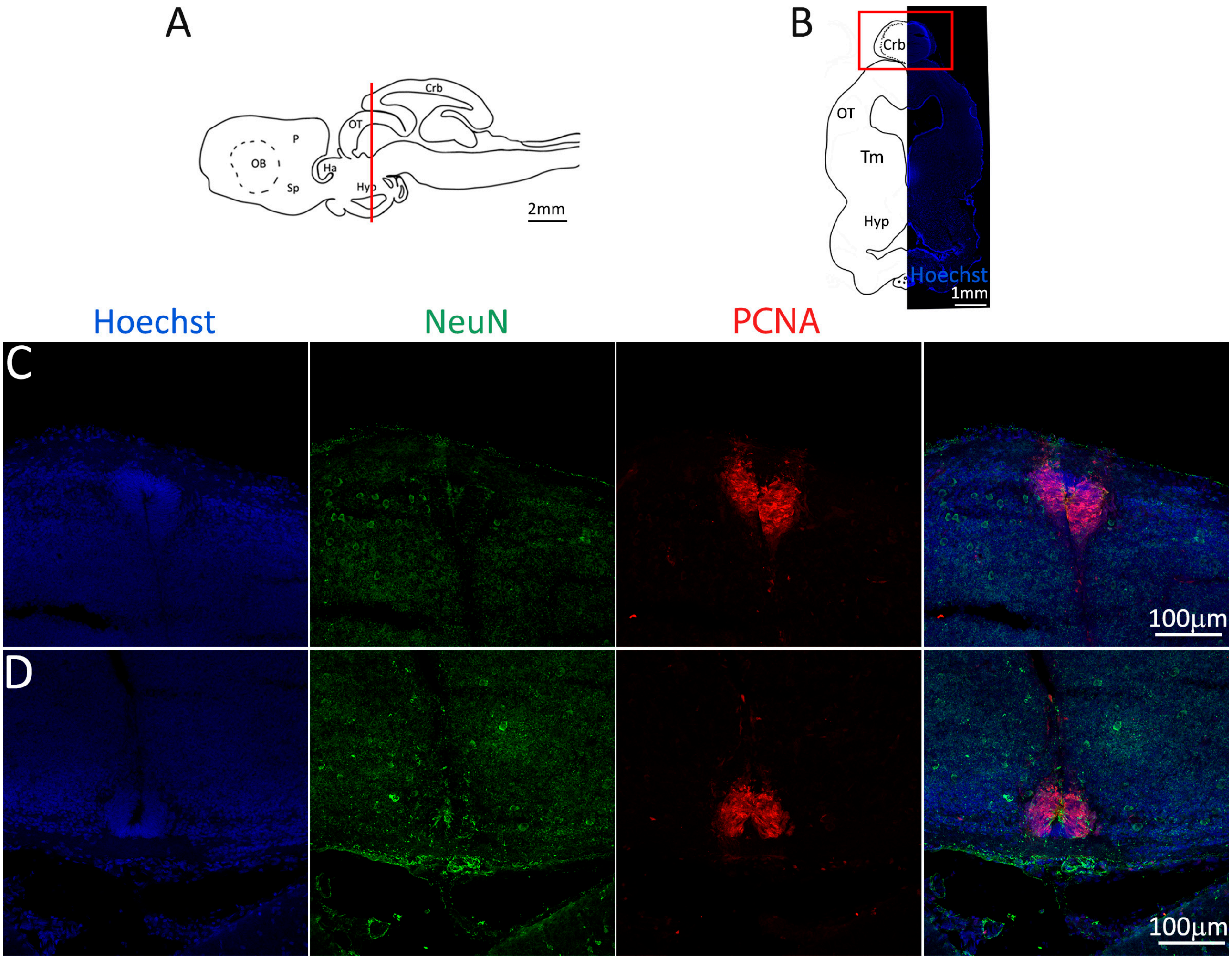

Figure S8. Localization of neuronal cells in the anterior cerebellum of *S. canicula*. A) Sagittal representation of *S. canicula* brain. The red line indicates the rostro-caudal localization of the section represented in the panel. B) Coronal map of the imaged area. On the right, a real coronal section is stained with Hoechst 33342 to show nuclei distribution, on the left a cartoon is drawn to indicate anatomical references. Red rectangle identifies the general area from which images are taken. C) Magnification of the neurogenic niche localized in the dorsal part of the anterior cerebellum. Neuronal cells (NeuN+, green) are located outside the neurogenic niche containing PCNA+ cells (red). D) Magnification of the neurogenic niche localized in the ventral portion of the anterior cerebellum. Neuronal cells (NeuN+, green) are located outside the neurogenic niche containing PCNA+ cells (red).

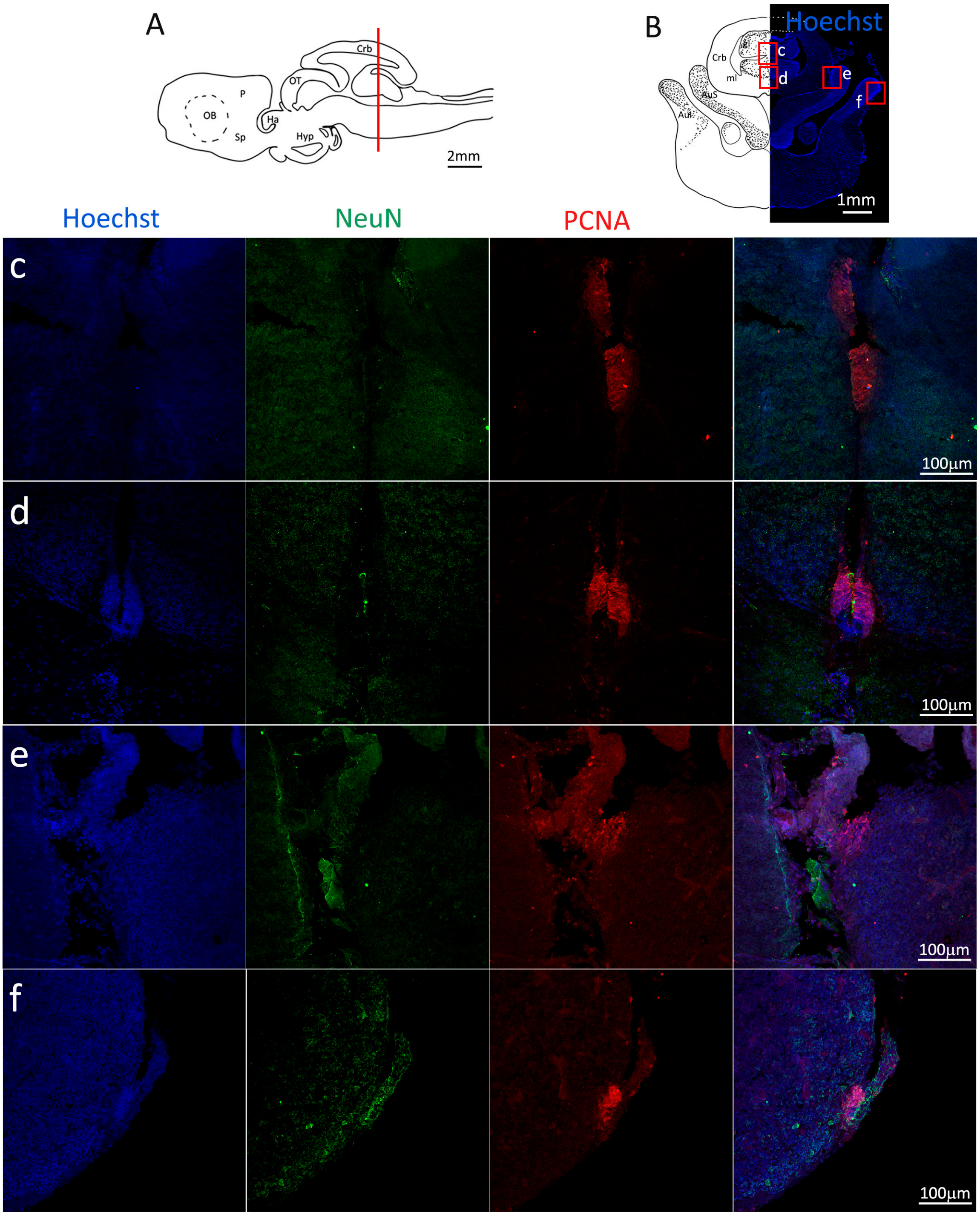

Figure S9. Localization of neuronal cells in the posterior cerebellum of *S. canicula*. A) Sagittal representation of *S. canicula* brain. The red line indicates the rostro-caudal localization of the section represented in the panel. B) Coronal map of the imaged area. On the right, a real coronal section is stained with Hoechst 33342 to show nuclei distribution, on the left a cartoon is drawn to indicate anatomical references. Red rectangles identify the general area from which images are taken. c) Magnification of the neurogenic niche localized in the dorsal part of the cerebellum. Neuronal cells (NeuN$^+$, green) are located outside the neurogenic niche containing PCNA$^+$ cells (red). d) Magnification of the neurogenic niche localized in the ventral portion of the cerebellum. Neuronal cells (NeuN$^+$, green) are located outside the neurogenic niche containing PCNA$^+$ cells (red). e) Magnification of the neurogenic niche localized in the dorsal cerebellar auricle. Neuronal cells (NeuN$^+$, green) are located outside the neurogenic niche containing PCNA$^+$ cells (red). f)

Magnification of the neurogenic niche localized in the ventral cerebellar auricle. Neuronal cells (NeuN⁺, green) are located outside the neurogenic niche containing PCNA⁺ cells (red).

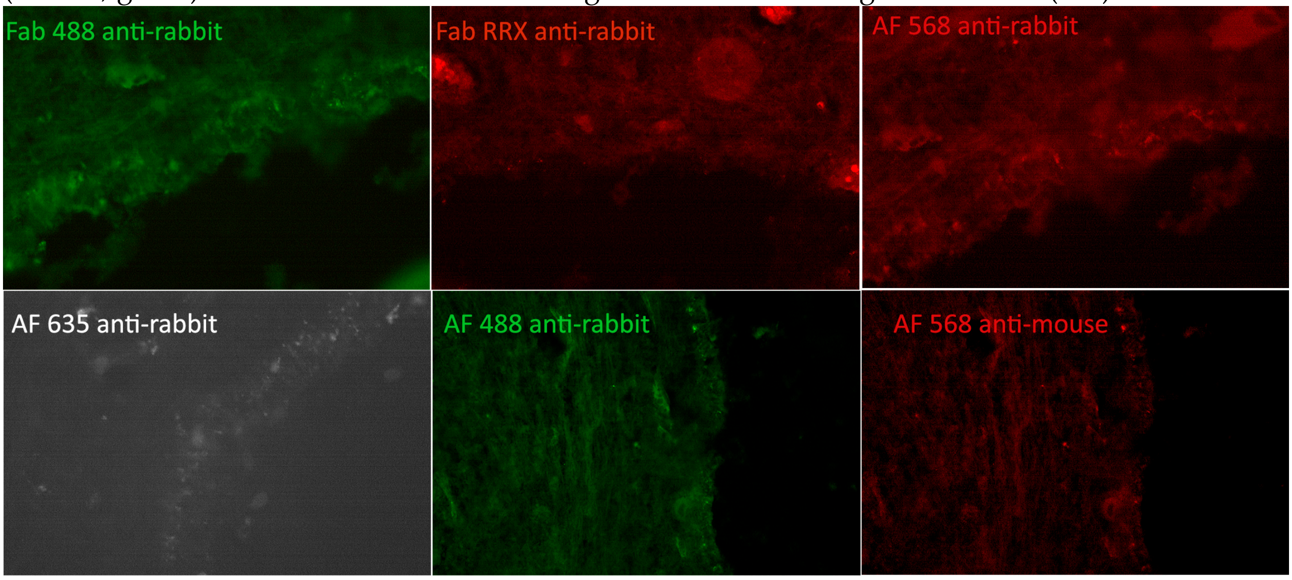

Figure S10. Negative controls for immunofluorescence experiments. In each panel, we report images of *S. canicula* brain tissue sections treated by full immunofluorescence procedure without applying the primary antibody and added with the respective secondary antibody. We imaged areas that, in fully traded samples, present clear staining for neurogenic niches.
